# Estudo sobre os Níveis Urinários de Cafeína e seus Metabólitos em Adultos Americanos com Doença Cardíaca Coronária: Estudo Transversal NHANES (2009-2014)

**DOI:** 10.36660/abc.20240425

**Published:** 2025-06-04

**Authors:** Qianqian Li, Gan Ke, Mengdie Liu, Yanjun Chen, Ye Luo, Liangxian Qiu

**Affiliations:** 1 Shenzhen University Shenzhen China Shenzhen University, Shenzhen – China; 2 Peking University Shenzhen Hospital Department of Cardiovascular Medicine Shenzhen China Peking University Shenzhen Hospital – Department of Cardiovascular Medicine, Shenzhen – China

**Keywords:** Cafeína, Doenças Cardiovasculares, Grupos Raciais

## Abstract

**Fundamento:**

Nos últimos anos, o impacto da dieta e do estilo de vida na doença cardíaca coronária (DCC) recebeu ampla atenção. A cafeína, um componente alimentar comum, e seus metabólitos podem estar relacionados à saúde cardiovascular.

**Objetivo:**

O estudo tem como objetivo investigar a associação entre os níveis urinários de cafeína e seus metabólitos e a incidência de DCC e avaliar a relação entre os níveis urinários de cafeína e a incidência de DCC em diferentes populações.

**Métodos:**

Os dados foram analisados usando modelos de regressão logística multivariável e métodos de ajuste de curva suave. Etapas específicas incluíram extrair dados relevantes do banco de dados NHANES de 2009 a 2014; categorizar os participantes em um grupo de controle (n = 5005) e um grupo DCC (n = 222) com base em seu status DCC; medir e registrar os níveis urinários de cafeína e seus metabólitos; aplicar modelos de regressão logística multivariável para avaliar a associação entre os níveis urinários de cafeína e seus metabólitos e a incidência de DCC; e usar métodos de ajuste de curva suave para explorar ainda mais as relações não lineares dessas associações. O nível de significância foi definido em 5% na análise estatística para determinar a significância estatística dos resultados.

**Resultados:**

Os níveis de paraxantina urinária foram inversamente relacionados ao risco de DCC. Os níveis de cafeína na urina foram positivamente associados à incidência de DCC em mexicano-americanos, mas essa associação não foi observada em outras populações.

**Conclusão:**

Níveis mais baixos de paraxantina urinária podem indicar um risco reduzido de DCC, enquanto níveis mais altos de cafeína na urina podem estar associados ao aumento do risco de DCC em populações específicas.

## Graphical abstract

**Figura Central:**
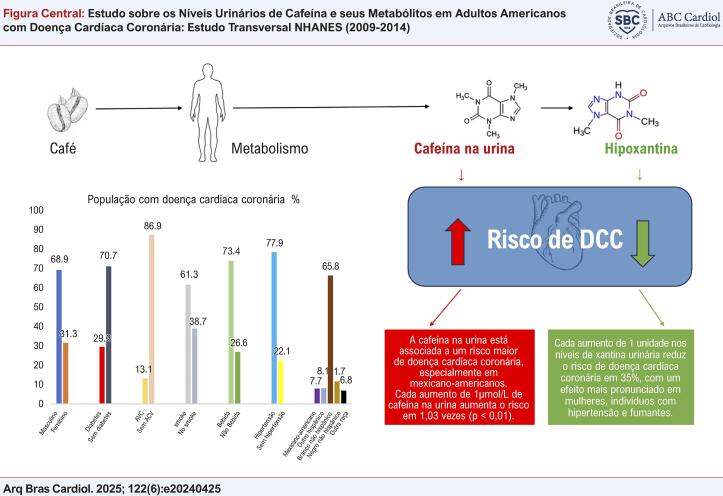
Estudo sobre os Níveis Urinários de Cafeína e seus Metabólitos em Adultos Americanos com Doença Cardíaca Coronária: Estudo Transversal NHANES (2009-2014)

## Histórico e importância da pesquisa

Nos últimos anos, a doença cardíaca coronária (DCC) se tornou uma das principais causas de morte, sendo responsável por mais de 370.000 mortes anualmente.^1^ Junto com fatores de risco tradicionais, como tabagismo, hiperlipidemia, hipertensão, diabetes e obesidade, novos fatores de risco foram identificados, incluindo inflamação sistêmica, diabetes gestacional, pré-eclâmpsia e menopausa precoce ou cirúrgica.

Foi demonstrado que modificações dietéticas reduzem a incidência de DCC,^2^ com compostos farmacológicos naturais, especialmente cafeína, frequentemente usados como terapias complementares para doenças cardiovasculares.^3^ O café, a bebida mais consumida globalmente, contém cafeína (1,3,7-trimetilxantina), que é ingerida por meio de alimentos e bebidas.^4 , 5^ Embora a cafeína possa causar um aumento temporário na pressão arterial em bebedores não habituais, o consumo de café a longo prazo pode ajudar a prevenir arritmias e insuficiência cardíaca e melhorar a resistência à insulina, reduzindo assim o risco de diabetes.

Estudos demonstraram que o consumo de café está inversamente relacionado à mortalidade por todas as causas, e o consumo habitual de café está associado à redução de riscos de doenças cardiovasculares e resultados cardiovasculares adversos, incluindo DCC, insuficiência cardíaca congestiva e acidente vascular cerebral (AVC). O consumo prolongado de café também está associado a menores riscos de diabetes tipo 2, depressão, obesidade e câncer.^6 , 7^ Especificamente, níveis aumentados de cafeína e teofilina na urina estão negativamente correlacionados com doenças cardiovasculares,^8^ com essa associação sendo mais pronunciada em mulheres.^9^ No entanto, a maioria das pesquisas atuais é baseada em pesquisas sobre hábitos de consumo de café, e diferentes marcas, origens e tipos de café contêm quantidades variadas de cafeína. Poucos estudos examinaram a relação entre metabólitos de cafeína e doenças cardiovasculares, e as pesquisas existentes frequentemente ignoram as diferenças causadas pelo tipo de café, origem e processos de produção, com potenciais erros em pesquisas por questionário impactando os resultados.

Um indicador confiável da ingestão de café é a quantidade de cafeína e seus metabólitos excretados na urina. Diferenças individuais no metabolismo da cafeína podem levar a variações nas concentrações circulantes de cafeína e metabólitos. Para entender melhor a associação entre cafeína na urina e seus metabólitos e DCC, conduzimos um estudo transversal usando dados da Pesquisa Nacional de Exame de Saúde e Nutrição (NHANES) de 2009-2014 para explorar possíveis relações entre cafeína, seus metabólitos e incidência de DCC.

## Materiais e métodos

### População do estudo

NHANES é uma pesquisa nutricional transversal nos Estados Unidos que usa um método de amostragem de probabilidade estratificada em vários estágios e foi aprovada pelo *National Center for Health Statistics* para coletar e disseminar dados.^10^ Este estudo selecionou dados relevantes de adultos (idade ≥ 20 anos) que participaram do NHANES de 2009 a 2014 para inclusão no estudo.^11^ Os autores não tiveram acesso a informações que pudessem identificar participantes individuais durante ou após a coleta de dados.

**Figura 1:**
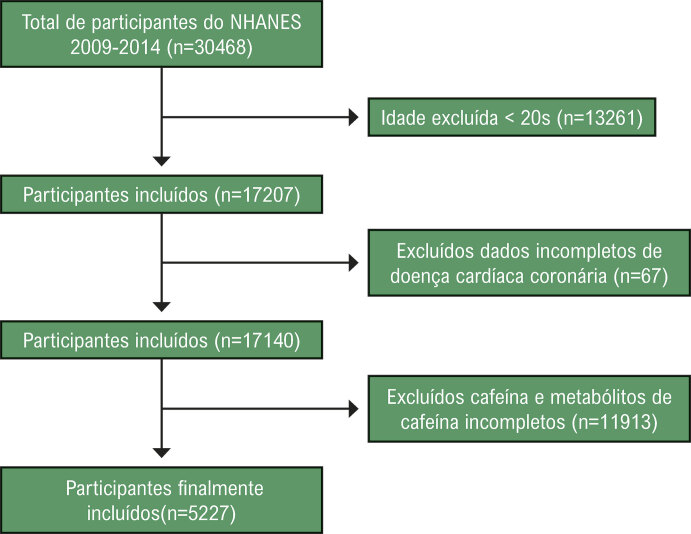
– Fluxograma de seleção de participantes.

Dados de um total de 30.468 participantes entre 2009 e 2014 foram inicialmente incluídos neste estudo. Os participantes deste estudo tinham menos de 20 anos (n = 13.261), tinham dados incompletos sobre DCC (n = 67) e dados incompletos sobre cafeína e metabólitos de cafeína (n = 11.913) foram excluídos, resultando em 5.227 indivíduos incluídos ( Figura 1 ).

### Fatores de exposição

Como a teobromina (3,7-dimetilxantina), a teofilina (1,3-dimetilxantina) e a paraxantina (1,7-dimetilxantina) são os principais metabolizadores de cafeína em substâncias humanas,^12^ portanto, neste estudo, os níveis urinários de cafeína e metabólitos de cafeína foram usados como variáveis de exposição. Amostras de urina foram coletadas entre 22 e 24 da manhã após os participantes do estudo passarem por um jejum noturno de 9 horas. As amostras de urina foram então recebidas, armazenadas e enviadas para a Divisão de Ciências Laboratoriais do Centro de Controle e Prevenção de Doenças no Centro Ambiental Nacional em Atlanta, Geórgia para a verificação. Finalmente, a cromatografia líquida de alta eficiência-ionização por eletrospray-espectrometria de massa quadrupolo em tandem (HPLC-ESI-MS/MS) e padrões internos marcados com isótopos estáveis foram usados para determinar a cafeína e seus metabólitos na urina,^13^ e os dados foram revisados. O laboratório responsável recebe valores incertos ou dados incompletos para verificação.

### Variável de resultado

O diagnóstico de DCC é baseado nos registros clínicos do paciente e nos resultados de exames de imagem (como angiografia coronária) e é coletado por entrevistadores experientes usando tecnologia de entrevista pessoal auxiliada por computador (CAPI). O questionário está disponível no site da NHANES.

### Covariáveis

Este estudo incluiu covariáveis que podem influenciar a relação entre cafeína e metabólitos de cafeína e DCC, incluindo gênero, idade, raça, histórico de pressão alta, histórico de colesterol alto, histórico de diabetes, histórico de AVC e tabagismo por pelo menos 100 anos na vida. Dados como gastos (%), consumo de pelo menos 12 bebidas alcoólicas/ano, MET (estado de atividade física) e IMC (índice de massa corporal) (kg/m^2^).^11^ Entre eles, o IMC é calculado pelo quadrado do peso/altura. Técnicos de saúde qualificados em centros de exames móveis coletaram medidas corporais. Outras covariáveis foram coletadas como variáveis de resultado por meio do sistema CAPI.

### Análise estatística

Usamos o software R e o Empower Statistics para análise estatística.^14^ Quando o valor-p do resultado da análise < 0,05, o resultado foi considerado significativo. Variáveis contínuas ausentes entre os indivíduos incluídos foram excluídas, enquanto variáveis categóricas ausentes formaram um grupo separado. O teste de normalidade para todas as variáveis contínuas é conduzido usando o teste de Kolmogorov-Smirnov, combinado com a visualização do histograma para análise. Se os dados seguirem uma distribuição normal, eles serão representados pela média ± desvio padrão, e testes-t de amostra independente serão usados para comparações de grupo. Se os dados não seguirem uma distribuição normal, eles serão representados pela mediana e intervalo interquartil (Q1-Q3), e o teste U de Mann-Whitney será usado para comparações de grupo. Após o teste de normalidade, descobrimos que os dados contínuos não aderem a uma distribuição normal (veja o Apêndice 1 para detalhes). Para variáveis contínuas com uma distribuição não normal, os dados são resumidos usando a mediana e o intervalo interquartil (Q1-Q3), e as comparações de grupo são conduzidas usando o teste U de Mann-Whitney. Os dados categóricos foram descritos como frequências absolutas (n) e relativas (%), e o teste qui-quadrado foi realizado para comparar as diferenças entre os grupos. Quando a frequência esperada é menor que 10, o teste exato de Fisher é usado. A análise de regressão logística multivariável avaliou a relação entre os níveis urinários de cafeína e metabólitos de cafeína e a DCC. Para dados contínuos com distribuição não normal, usamos o método de agrupamento de quartis (Q1, Q2, Q3, Q4) para dividir os dados em quatro partes, fornecendo uma melhor representação de sua distribuição. Q4 representa o quarto dos dados, não o quinto segmento de uma análise de quintil. Em nosso estudo, o conjunto de dados é dividido em quatro quartis (Q1, Q2, Q3, Q4) com base nos valores da variável de interesse. Q1 representa os 25% mais baixos dos dados, Q2 representa os 50% do meio e Q3 representa os 25% superiores. Q4, portanto, representa o quartil final, que inclui os 25% superiores dos dados, estendendo-se de Q3 até o valor máximo. É importante observar que Q4 não é um ponto limite adicional, mas o intervalo mais alto dos dados, cobrindo os 25% finais. Ao usar o agrupamento de quartis Q1-Q4, podemos demonstrar claramente a tendência da variável de interesse em diferentes intervalos de distribuição de dados. Especificamente, Q1 representa os 25% mais baixos dos dados, fornecendo uma linha de base para comparações com os quartis mais altos (Q2, Q3, Q4). Comparado aos grupos Q2, Q3 e Q4, podemos observar mais claramente as diferenças entre diferentes níveis de distribuição. Q4, representando os 25% superiores dos dados, nos permite destacar as características do grupo de alto valor e suas diferenças de outros grupos. Este método de agrupamento ajuda a revelar melhor as tendências e mudanças na distribuição de dados, oferecendo uma perspectiva analítica mais detalhada. Escolhemos Q1 (os 25% mais baixos) como o grupo “Referência” porque ele representa a população de baixo risco/baixo nível, tornando-o adequado como um grupo de controle. Neste agrupamento, os outros grupos (Q2, Q3, Q4) representam níveis progressivamente mais altos, o que ajuda a demonstrar claramente as diferenças entre os grupos de nível inferior e superior.

Este estudo criou um total de 3 modelos relacionados, primeiro criando um modelo 1 sem ajustar variáveis, depois criando um modelo ajustado 2 usando variáveis incluindo gênero, idade e raça ou etnia, e finalmente criando um modelo ajustado 2 ajustando gênero, idade, raça, altura. O modelo 3 foi obtido com base no histórico de pressão arterial, colesterol alto, diabetes, AVC, tabagismo de pelo menos 100 cigarros na vida (%), consumo de pelo menos 12 bebidas alcoólicas/ano e IMC (kg/m^2^). A análise de várias covariáveis com base nos três modelos acima esclareceu ainda mais a relação entre cafeína na urina e seus metabólitos e DCC. O ajuste de curva suave foi realizado para investigar melhor a relação entre cafeína e níveis de metabólitos de cafeína e DCC após controlar algumas variáveis. Além disso, também conduzimos análise de subgrupos para explorar mais as associações entre variáveis e identificar as características específicas de diferentes subgrupos segmentando a população.

### Aprovação ética

Os dados para este estudo vieram do site da NHANES. Portanto, não requer aprovação do conselho de revisão institucional (ou comitê de ética).

## Resultados

### Características básicas da população da pesquisa

O teste de normalidade revelou que os dados contínuos não seguiam uma distribuição normal (ver Apêndice 1). As variáveis contínuas com distribuição não normal são apresentadas como mediana e intervalo interquartil, com as diferenças entre os grupos avaliadas pelo teste de Mann-Whitney U. As variáveis categóricas são expressas como N (%), e as diferenças entre os grupos são analisadas utilizando o teste do qui-quadrado ou o teste exato de Fisher quando as frequências esperadas são inferiores a 10. Um valor de p < 0,05 indica significância estatística.

Os participantes deste estudo foram divididos em um grupo de controle (n = 5005) e um grupo de DCC (DCC) (n = 222). A análise revelou diferenças significativas nas características basais entre os dois grupos, com exceção do IMC, cafeína na urina e metabólitos da cafeína — por exemplo, idade, sexo, raça, escore MET etc. Notavelmente, os indivíduos no grupo de DCC eram significativamente mais velhos (72 (IQR: 63-80) anos vs. 48 (IQR: 34-62) anos, p < 0,001) e exibiram uma prevalência maior de hipertensão, hipercolesterolemia, diabetes, AVC, bem como histórico de tabagismo e bebida (todos p < 0,001). Além disso, houve diferenças observáveis na distribuição étnica entre os dois grupos (p = 0,001),

Houve diferença significativa nos níveis de paraxantina entre os dois grupos (p = 0,018), conforme resumido na Tabela 1 .

**Tabela 1:** – Características básicas e níveis de cafeína e metabólitos de cafeína na urina dos participantes do estudo

Variáveis	Grupo de doenças cardíacas (n=222)	Grupo de controle (n=5005)	Diferença padronizada	Valor-p	Valor-p*
**IDADE (anos)**	72(63-80)	48(34-62)	1,5 (1,3; 1,6)	<0,001	
**SEXO**			0,4 (0,3; 0,6)	<0,001	-
Masc.	153 (68,9%)	2390 (47,8%)			
Fem.	69 (31,1%)	2615 (52,2%)			
**RAÇA**			0,5 (0,4; 0,6)	<0,001	-
Mexicano-americano	17 (7,7%)	700 (14,0%)			
Outro hispânico	18 (8,1%)	504 (10,1%)			
Branco não hispânico	146 (65,8%)	2111 (42,2%)			
Negro não hispânico	26 (11,7%)	1074 (21,5%)			
Outra raça - incluindo multirracial	15 (6,8%)	616 (12,3%)			
**PRESSÃO ALTA**			1,0 (0,8; 1,1)	<0,001	
Sim	173 (77,9%)	1735 (34,7%)			
Não	48 (21,6%)	3262 (65,2%)			
Não sabe	1 (0,5%)	8 (0,2%)			
**NÍVEL ALTO DE COLESTEROL**			1,0 (0,9; 1,1)	<0,001	-
Sim	163 (73,4%)	1552 (31,0%)			
Não	56 (25,2%)	2887 (57,7%)			
Não sabe	3 (1,4%)	31 (0,6%)			
Ausente	0 (0,0%)	535 (10,7%)			
**DIABETES**			0,5 (0,4; 0,6)	<0,001	-
Sim	65 (29,3%)	550 (11,0%)			
Não	147 (66,2%)	4333 (86,6%)			
Limite	10 (4,5%)	117 (2,3%)			
Não sabe	0 (0,0%)	5 (0,1%)			
**AVC**			0,4 (0,3; 0,5)	<0,001	
Sim	29 (13,1%)	138 (2,8%)			
Não	192 (86,5%)	4864 (97,2%)			
Não sabe	1 (0,5%)	3 (0,1%)			
**FUMOU PELO MENOS 100 CIGARROS NA VIDA**			0,4 (0,2; 0,5)	<0,001	
Sim	136 (61,3%)	2160 (43,2%)			
Não	86 (38,7%)	2844 (56,8%)			
Não sabe	0 (0,0%)	1 (0,0%)			
**PELO MENOS 12 BEBIDAS ALCOÓLICAS POR 1 ANO**			0,2 (0,0; 0,3)	0,195	-
Sim	156 (70,3%)	3278 (65,5%)			
Não	54 (24,3%)	1249 (25,0%)			
Não sabe	0 (0,0%)	2 (0,0%)			
Ausente	12 (5,4%)	476 (9,5%)			
**MET**			0,4 (0,3; 0,6)	<0,001	-
<4	91 (41,0%)	1264 (25,3%)			
4-8	70 (31,5%)	1392 (27,8%)			
≥8	61 (27,5%)	2349 (46,9%)			
**IMC (kg/m** ^**2**^ **)**	28,86 (25.803; 32.975)	28(24,3; 32,42)	0,2 (0,0; 0,3)	0,023	0,007
**TEOFILINA (μmol/L)**	1,645 (0,625; 3,538)	1,88(0,639; 3,84)	0,1 (-0,1; 0,2)	0,532	0,367
**PARAXANTINA (μmol/L)**	16,6 (4,738; 29,325)	17,5 (5,72; 37,4)	0,2 (0,0; 0,3)	0,018	0,098
**TEOBROMINA (μmol/L)**	13,5 (6,285; 29,8)	15,3(6,36; 34)	0,1 (-0,1; 0,2)	0,367	0,289
**CAFEÍNA (μmol/L)**	5,465(1,533; 12,2)	4,23(1,01; 10,8)	0,1 (-0,0; 0,2)	0,086	0,045

AVC: acidente vascular cerebral; IMC: índice de massa corporal. O teste de normalidade revelou que os dados contínuos não seguiram uma distribuição normal (ver Apêndice 1). Variáveis contínuas não distribuídas normalmente são apresentadas como mediana e intervalo interquartil, com diferenças de grupo avaliadas usando o teste U de Mann-Whitney. Variáveis categóricas são expressas como N (%), e diferenças de grupo são analisadas usando o teste qui-quadrado ou exato de Fisher quando as frequências esperadas são abaixo de 10. Um valor de p < 0,05 indica significância estatística.

### A relação entre os níveis de cafeína na urina e a doença cardíaca coronária

Os resultados da relação entre os níveis de cafeína na urina e a DCC são mostrados na Tabela 2 . Primeiro, sem ajuste para diferentes covariáveis, os níveis de cafeína na urina foram consistentes com a DCC sem significância estatística (todos p > 0,05). Os níveis contínuos de cafeína foram então convertidos em variáveis categóricas (quartis) dos quatro subgrupos, e pode-se ver que o nível de cafeína na urina no grupo Q4 foi positivamente associado à incidência de DCC (razão de chances [OR] = 1,54, intervalo de confiança [IC] de 95%: (1,04, 2,3), p = 0,0318). Ou seja, para cada aumento de unidade (μmol/L) no nível de cafeína na urina, o risco de DCC é 1,54 vezes maior do que antes do aumento.

**Tabela 2:** – Relação entre níveis urinários de cafeína (μmol/L) e doença cardíaca coronária. Modelo 1: Covariáveis não ajustadas. Modelo 2: Ajuste para idade, gênero e raça dos indivíduos. Modelo 3: Ajuste para idade, gênero, raça, pressão alta, colesterol alto, diabetes, AVC, tabagismo, bebida e IMC.

	Modelo 1 OR (IC 95%) Valor - p	Modelo 2 OR (IC 95%) Valor - p	Modelo 3 OR (IC 95%) Valor - p
**Cafeína (μmol/L)**	1,01 (1,00, 1,02) 0,0859	1,00 (0,99, 1,01) 0,9476	0,99 (0,98, 1,01) 0,3973
**Categorias de cafeína**			
Q1	Referência	Referência	Referência
Q2	1,37 (0,91, 2,06) 0,1257	1,20 (0,78, 1,85) 0,3998	1,13 (0,73, 1,77) 0,5799
3º trimestre	1,42 (0,95, 2,13) 0,0874	1,18 (0,77, 1,81) 0,4479	1,01 (0,65, 1,59) 0,9487
4º trimestre	1,54 (1,04, 2,30) 0,0318	1,10 (0,72, 1,68) 0,6571	0,94 (0,61, 1,47) 0,8006
**Sexo**			
Masc.	1,0 (1,0, 1,0) 0,009	1,0 (1,0, 1,0) 0,545	1,0 (1,0, 1,0) 0,804
Fem.	1,0 (1,0, 1,0) 0,721	1,0 (1,0, 1,0) 0,282	1,0 (1,0, 1,0) 0,155
**Raça**			
Mexicano-americano	1,03 (1,01, 1,05) 0,0081	1,03 (1,00, 1,05) 0,0700	1,02 (0,98, 1,06) 0,2327
Outro hispânico	1,03 (0,99, 1,07) 0,1409	1,02 (0,97, 1,06) 0,4538	1,00 (0,95, 1,05) 0,9737
Branco não hispânico	0,99 (0,98, 1,01) 0,3734	0,99 (0,97, 1,01) 0,2670	0,98 (0,97, 1,00) 0,0826
Negro não hispânico	1,00 (0,95, 1,05) 0,9936	0,99 (0,94, 1,04) 0,6900	0,99 (0,93, 1,04) 0,6351

**Figura 2:**
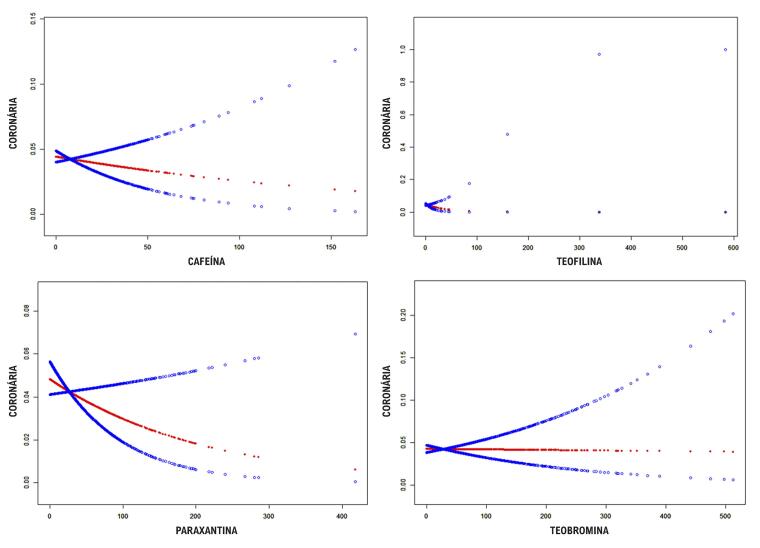
– Ajuste de curva suave da relação entre os níveis urinários de cafeína e metabólitos de cafeína (μmol/L) e a incidência de doença cardíaca coronária. A linha vermelha representa o ajuste de curva suave entre cafeína e metabólitos de cafeína e doença cardíaca coronária. A linha azul representa seu intervalo de confiança de 95%. Ajustado para gênero, idade, raça, pressão alta, colesterol alto, diabetes, AVC, tabagismo, consumo de álcool e IMC do participante.

As análises foram então estratificadas por gênero e raça. Em análises estratificadas por sexo, os níveis de cafeína urinária não foram associados à incidência de DCC (razão de chances [OR] = 1,0, intervalo de confiança [IC] de 95%: (1,0, 1,0), p = 0,009). Em uma análise de subgrupo étnico, os níveis de cafeína na urina foram positivamente associados à incidência de DCC em mexicano-americanos (razão de chances [OR] = 1,03, intervalo de confiança [IC] de 95%: (1,03, 1,05), p = 0,0081), ou seja, para cada aumento de unidade (μmol/L) no nível de cafeína na urina, o risco de DCC aumenta em 1,03 vezes. Os resultados de outros subgrupos mostraram que nenhuma relação significativa foi observada entre os metabólitos da cafeína na urina e a DCC (todos p > 0,05). A Figura 2A mostra um ajuste de curva suave da relação entre os níveis de cafeína na urina e a incidência de DCC.

### Associação entre os níveis de metabólitos urinários de cafeína e doença cardíaca coronária

Os resultados da relação entre os níveis de metabólitos de cafeína na urina e a DCC são mostrados na Tabela 3 . Primeiro, sem ajuste para diferentes covariáveis, os níveis de teofilina e teobromina na urina foram consistentes com a DCC e não tiveram significância estatística (todos p > 0,05). Os níveis contínuos de teofilina e teobromina na urina foram então convertidos em variáveis categóricas (quartis). Nenhuma é estatisticamente significativa. Após o ajuste das covariáveis, não houve significância estatística entre os níveis de teofilina e teobromina na urina e a DCC (todos p > 0,05). Um ajuste de curva suave da relação entre os níveis de teofilina e teobromina na urina e a incidência de DCC é mostrado nas Figuras 2B e C .

**Tabela 3:** – Relação entre os níveis de metabólitos de cafeína na urina (μmol/L) e doença cardíaca coronária. Modelo 1: Covariáveis não ajustadas. Modelo 2: Ajuste para idade, gênero e raça dos indivíduos. Modelo 3: Ajuste para idade, gênero, raça, pressão alta, colesterol alto, diabetes, AVC, tabagismo, bebida alcoólica e IMC

	Modelo 1 OR (IC 95%) Valor - p	Modelo 2 OR (IC 95%) Valor - p	Modelo 3 OR (IC 95%) Valor - p
**Teofilina (μmol/L)**	0,98 (0,93, 1,02) 0,3320	0,98 (0,94, 1,02) 0,3084	0,97 (0,93, 1,02) 0,2592
**Categorias de Teofilina**			
Q1	Referência	Referência	Referência
Q2	1,10 (0,76, 1,59) 0,6243	1,00 (0,67, 1,49) 0,9914	0,94 (0,62, 1,42) 0,7712
3º trimestre	0,94 (0,64, 1,38) 0,7660	0,89 (0,59, 1,34) 0,5788	0,83 (0,54, 1,27) 0,3800
4º trimestre	0,92 (0,63, 1,36) 0,6883	0,90 (0,59, 1,36) 0,6020	0,85 (0,55, 1,31) 0,4527
**Teobromina (μmol/L)**	1,00 (0,99, 1,00) 0,3674	1,00 (1,00, 1,00) 0,6186	1,00 (1,00, 1,00) 0,9285
**Categorias de Teobromina**			
Q1	Referência	Referência	Referência
Q2	1,12 (0,77, 1,62) 0,5605	1,19 (0,80, 1,76) 0,3965	1,09 (0,73, 1,64) 0,6724
3º trimestre	1,09 (0,75, 1,58) 0,6515	1,14 (0,76, 1,70) 0,5217	1,10 (0,73, 1,68) 0,6430
4º trimestre	0,76 (0,51, 1,14) 0,1816	0,84 (0,54, 1,30) 0,4293	0,89 (0,57, 1,40) 0,6291
**Paraxantina (μmol/L)**	0,99 (0,99, 1,00) 0,0177	1,00 (0,99, 1,00) 0,1037	0,99 (0,99, 1,00) 0,1154
**Categorias de Paraxantina**			
Q1	Referência	Referência	Referência
Q2	0,90 (0,62, 1,31) 0,5866	0,76 (0,51, 1,13) 0,1789	0,74 (0,49, 1,11) 0,1461
3º trimestre	1,01 (0,71, 1,45) 0,9409	0,99 (0,67, 1,45) 0,9396	0,97 (0,65, 1,45) 0,8707
4º trimestre	0,65 (0,43, 0,97) 0,0360	0,68 (0,44, 1,04) 0,0779	0,65 (0,42, 1,03) 0,0655

Ajuste de curva suave da relação entre os níveis urinários de cafeína e metabólitos de cafeína (μmol/L) e a incidência de doença cardíaca coronária

Os resultados do modelo 1 mostraram que níveis elevados de paraxantina urinária estavam associados a uma incidência reduzida de DCC (OR = 0,99, IC 95% (0,99, 1,0), p = 0,0177). Os modelos 2 e 3 não mostraram nenhuma relação significativa entre os níveis de paraxantina urinária e a DCC após o ajuste para covariáveis. Para análise de regressão multivariável, transformamos ainda mais os níveis de paraxantina urinária de uma variável contínua em uma variável categórica (quatro categorias). No grupo com os maiores níveis de paraxantina urinária (Q4), os níveis de paraxantina urinária foram inversamente associados à redução da incidência de DCC no modelo 1 (OR = 0,65, IC 95% (0,43, 0,97), p = 0,036), mas não houve significância estatística em outros modelos (todos p > 0,05).

### Diferenças nos níveis urinários de cafeína e metabólitos de cafeína (μ mol/L) entre o grupo controle e o grupo com doença cardíaca coronária

Explore as diferenças nas concentrações de teofilina, paraxantina, teobromina e cafeína entre o grupo de doença cardíaca e o grupo de controle. O estudo usou modelos não ajustados, Ajuste I e Ajuste II para análise. A tabela lista quatro compostos - Teofilina, Paraxantina, Teobromina e Cafeína. Dividida no grupo de controle, a população sem doença cardíaca é definida como o grupo de controle, com um tamanho de amostra de 5005, e o grupo de doença cardíaca, a população com doença cardíaca, com um tamanho de amostra de 222. A comparação de cada composto entre o grupo de doença cardíaca e o grupo de controle é representada pela diferença (intervalo de confiança de 95%) e valor - p: a diferença é a diferença de concentração média entre o grupo de doença cardíaca e o grupo de controle. O intervalo de confiança de 95% (entre parênteses) representa o intervalo estimado de diferenças. O valor - p é usado para testar se a diferença é estatisticamente significativa (valor de p < 0,05 é considerado estatisticamente significativo).

Os resultados apresentados na Tabela 4 indicam que a Teofilina Não Ajustada teve uma diferença de -0,4 (-0,9, 0,1), p=0,0923, que não atingiu significância estatística. Ajuste I: A diferença é -0,8 (-1,5, -0,1), p=0,0196, significativo. Ajuste II: A diferença é -0,7 (-1,5, -0,0), p=0,0412, significativo. Após o ajuste, a concentração de teofilina no grupo com doença cardíaca foi significativamente menor do que no grupo controle. Paraxantina Não Ajustada: A diferença é -4,8 (-8,0, -1,7), p=0,0028, significativo. Ajuste I: A diferença é -5,1 (-8,4, -1,8), p=0,0023, significativo. Ajuste II: A diferença é -4,1 (-7,3, -0,8), p=0,0135, significativo. Os níveis de paraxantina foram significativamente menores em todos os modelos em comparação ao grupo de controle. Teobromina Não ajustado: A diferença é -2,5 (-8,2, 3,2), p=0,3925, não significativo. Ajuste I: A diferença é -0,1 (-5,9, 5,7), p=0,9689, não significativo. Ajuste II: A diferença é -0,1 (-5,9, 5,7), p=0,9770, não significativo. Não houve diferença significativa na concentração de teobromina entre o grupo com doença cardíaca e o grupo de controle. Cafeína Não ajustado: A diferença é 1,2 (-0,4, 2,8), p=0,1327, não significativo. Ajuste I: A diferença é -0,9 (-2,5, 0,8), p=0,2966, não significativo. Ajuste II: A diferença é -0,5 (-2,1, 1,1), p=0,5534, não significativo. Após o ajuste, não houve diferença significativa na concentração de cafeína entre os dois grupos. Portanto, podemos concluir que as concentrações de teofilina e hipoxantina no grupo com doença cardíaca foram significativamente menores do que aquelas no grupo controle, especialmente após o ajuste, que ainda mostrou significância estatística, sugerindo que esses compostos podem estar associados à doença cardíaca. Não houve diferença significativa nas concentrações de teobromina e cafeína entre os dois grupos, o que pode não estar diretamente relacionado à doença cardíaca. O impacto de diferentes modelos de ajuste nos resultados é limitado, mas os resultados ajustados são mais confiáveis.

**Tabela 4:** – Diferenças nos níveis urinários de cafeína e metabólitos de cafeína (μ mol/L) entre o grupo controle e o grupo com doença cardíaca coronária. Modelo 1: Covariáveis não ajustadas. Modelo 2: Ajuste para idade, gênero, raça, pressão alta, colesterol alto, diabetes, AVC, tabagismo, bebida e IMC. Modelo 3: Ajuste para idade (baixo, médio, alto), gênero, raça, pressão alta, colesterol alto, diabetes, AVC, tabagismo, bebida e IMC (baixo, médio, alto)

	Não ajustado	Ajuste I	Ajuste II
**TEOFILINA**			
Grupo de controle (n = 5005)	Ref.	Ref.	Ref.
Grupo de doenças cardíacas (n=222)	-0,4 (-0,9, 0,1) 0,0923	-0,8 (-1,5, -0,1) 0,0196	-0,7 (-1,5, -0,0) 0,0412
**PARAXANTINA**			
Grupo de controle (n = 5005)	Ref.	Ref.	Ref.
Grupo de doenças cardíacas (n=222)	-4,8 (-8,0, -1,7) 0,0028	-5,1 (-8,4, -1,8) 0,0023	-4,1 (-7,3, -0,8) 0,0135
**TEOBROMINA**			
Grupo de controle (n = 5005)	Ref.	Ref.	Ref.
Grupo de doenças cardíacas (n=222)	-2,5 (-8,2, 3,2) 0,3925	-0,1 (-5,9, 5,7) 0,9689	-0,1 (-5,9, 5,7) 0,9770
**CAFEÍNA**			
Grupo de controle (n = 5005)	Ref.	Ref.	Ref.
Grupo de doenças cardíacas (n=222)	1,2 (-0,4, 2,8) 0,1327	-0,9 (-2,5, 0,8) 0,2966	-0,5 (-2,1, 1,1) 0,5534

### Análise de subgrupos

Variáveis de subgrupo: incluindo sexo, idade, raça, pressão alta, nível alto de colesterol, diabetes, tercil de IMC, AVC, tabagismo, bebidas alcoólicas em 1 ano, escore MET (escore MET < 4: indica restrição funcional grave, que está relacionada a risco significativamente aumentado de doença cardiovascular. Escore MET 4-6: indica restrição funcional moderada, que pode ser dispneia significativa ou fadiga ao completar atividades de intensidade moderada (como caminhada de curta distância e trabalho leve). Escore MET > 8 geralmente indica um risco menor de doença cardiovascular. Tamanho da amostra (N): O número de amostras em cada subgrupo. Diferença de concentração e significância estatística: Em cada subgrupo, exiba a diferença (intervalo de confiança de 95% e valor de p) entre o grupo de doença cardíaca e o grupo de controle na concentração de quatro compostos.

Através da análise hierárquica, como mostrado na Tabela 5 , descobrimos que na maioria dos subgrupos, a diferença de concentração de teofilina não atingiu significância estatística (p > 0,05). Por exemplo, na população sem hipertensão, a diferença foi de -1,3 (IC 95%: -4,8, 2,2), mas ainda não significativa (p = 0,4758). Não houve diferença significativa na concentração de teofilina entre os grupos. A diferença geral em Paraxantina foi significativa e consistente em vários subgrupos: gênero: feminino (diferença -7,4, p = 0,0379), masculino (diferença -4,8, p = 0,0538). Raça: Branco não hispânico (diferença -7,3, p = 0,0034); Não houve diferença significativa em outros grupos. Pacientes hipertensos: A diferença é de -9,7, p = 0,0299. No grupo de IMC alto, a diferença é de -6,5, p = 0,0457. Sem histórico de tabagismo: diferença de -7,0, p = 0,0265, MET<4 pontos: a diferença é -6,7, p = 0,0362.

**Tabela 5:** – Análise de subgrupos. Esta tabela apresenta os resultados da análise estratificada de teofilina, paraxantina, teobromina e cafeína em diferentes subgrupos, incluindo valores de efeito, intervalos de confiança de 95% e valores - p. Por meio da análise estratificada, diferenças nas concentrações de compostos e sua significância podem ser observadas entre diferentes grupos característicos

Subgrupo	No.	TEOFILINA	PARAXANTINA	TEOBROMINA	CAFEÍNA
**SEXO**					
Masc.	2543	-0,4 (-2,7, 1,8) 0,7161	-4,8 (-9,8, 0,1) 0,0538	0,4 (-6,0, 6,8) 0,9105	2,2 (0,6, 3,8) 0,0081
Fem.	2684	-0,7 (-1,7, 0,4) 0,2290	-7,4 (-14,5, -0,4) 0,0379	-6,7 (-16,5, 3,1) 0,1791	-0,5 (-3,1, 2,1) 0,7220
**IDADE ANO Tercil**					
Baixo (18≤idade≤39)	1685	-0,9 (-3,6, 1,9) 0,5380	-11,0 (-37,5, 15,4) 0,4137	-17,7 (-53,9, 18,5) 0,3381	-3,9 (-11,3, 3,6) 0,3106
Médio (39<idade≤57)	1740	-0,6 (-1,7, 0,5) 0,2582	-2,0 (-13,3, 9,3) 0,7259	-8,8 (-23,3, 5,8) 0,2373	0,9 (-2,6, 4,4) 0,6177
Alto (58≤idade≤80)	1802	-0,8 (-3,3, 1,7) 0,5434	-3,6 (-7,7, 0,5) 0,0882	2,0 (-3,8, 7,8) 0,5072	0,3 (-1,5, 2,1) 0,7589
**RAÇA**					
Mexicano-americano	717	1,7 (-0,0, 3,4) 0,0531	3,1 (-12,4, 18,5) 0,6971	3,7 (-12,0, 19,4) 0,6447	9,0 (3,9, 14,1) 0,0006
Outro hispânico	522	0,0 (-1,4, 1,5) 0,9699	1,2 (-12,3, 14,8) 0,8565	-5,4 (-22,1, 11,3) 0,5295	3,3 (-1,0, 7,6) 0,1348
Branco não hispânico	2257	-1,1 (-3,6, 1,4) 0,3858	-7,3 (-12,2, -2,4) 0,0034	-4,9 (-12,3, 2,5) 0,1936	-0,9 (-2,8, 1,1) 0,3745
Negro não hispânico	1100	-0,7 (-1,6, 0,2) 0,1460	-7,7 (-18,3, 2,9) 0,1538	-7,7 (-22,1, 6,7) 0,2933	-0,0 (-3,1, 3,0) 0,9936
Outra raça - incluindo multirracial	631	-0,3 (-1,9, 1,2) 0,6714	-2,6 (-19,8, 14,6) 0,7695	-7,0 (-27,6, 13,6) 0,5071	1,7 (-2,8, 6,1) 0,4658
**PRESSÃO ALTA**					
Sim	1908	0,1 (-0,5, 0,7) 0,8032	-1,4 (-5,6, 2,9) 0,5379	1,5 (-4,2, 7,2) 0,6026	1,5 (-0,2, 3,3) 0,0869
Não	3310	-1,3 (-4,8, 2,2) 0,4758	-9,7 (-18,5, -1,0) 0,0299	-7,2 (-19,2, 4,7) 0,2365	-1,5 (-4,3, 1,3) 0,2977
Não sabe	9				
**NÍVEL ALTO DE COLESTEROL**					
Sim	1715	-0,2 (-0,7, 0,3) 0,4859	-3,7 (-8,3, 1,0) 0,1196	-1,6 (-8,3, 5,0) 0,6333	1,0 (-0,8, 2,8) 0,2794
Não	2943	-0,7 (-3,8, 2,4) 0,6428	-7,0 (-15,1, 1,1) 0,0924	-3,6 (-14,0, 6,7) 0,4927	-0,4 (-3,1, 2,4) 0,7814
Não sabe	34	-0,5 (-4,8, 3,9) 0,8378	-13,2 (-57,1, 30,7) 0,5605	-14,6 (-68,1, 38,9) 0,5969	0,0 (-12,4, 12,4) 0,9987
Ausente	535				
**DIABETES**					
Sim	615	-0,4 (-1,2, 0,4) 0,3501	-6,8 (-13,6, 0,0) 0,0513	0,1 (-8,5, 8,7) 0,9758	-1,0 (-3,6, 1,7) 0,4773
Não	4480	-0,2 (-1,8, 1,4) 0,7934	-2,6 (-7,6, 2,3) 0,2998	-0,9 (-7,5, 5,7) 0,7801	2,1 (0,4, 3,8) 0,0165
Limite	127	-3,2 (-22,6, 16,3) 0,7500	-8,0 (-28,2, 12,3) 0,4417	-11,2 (-50,9, 28,6) 0,5830	-0,6 (-9,3, 8,0) 0,8841
Não sabe	5				
**Tercil de IMC**					
Baixo (13,4≤IMC≤25,69)	1724	-1,0 (-5,1, 3,0) 0,6270	-5,9 (-14,0, 2,2) 0,1543	-2,6 (-13,4, 8,2) 0,6394	-0,6 (-3,2, 2,1) 0,6778
Médio (25,7≤IMC≤30,59)	1750	-0,2 (-2,1, 1,7) 0,8438	-2,9 (-9,6, 3,8) 0,3952	-0,5 (-9,6, 8,5) 0,9080	1,9 (-0,6, 4,3) 0,1384
Alto (30,7≤IMC≤82,1)	1753	-0,3 (-1,0, 0,4) 0,4031	-6,5 (-12,8, -0,1) 0,0457	-4,9 (-13,6, 3,7) 0,2616	1,5 (-0,7, 3,7) 0,1743
**AVC**					
Sim	167	-1,0 (-3,8, 1,7) 0,4676	-4,6 (-13,8, 4,6) 0,3249	-6,3 (-17,6, 5,0) 0,2746	-1,1 (-5,5, 3,3) 0,6228
Não	5056	-0,3 (-1,8, 1,1) 0,6445	-4,1 (-8,4, 0,3) 0,0663	-1,2 (-7,0, 4,6) 0,6889	1,3 (-0,2, 2,8) 0,0862
Não sabe	4				
**FUMOU PELO MENOS 100 CIGARROS NA VIDA**					
Sim	2296	-0,1 (-1,6, 1,4) 0,8906	-4,8 (-10,1, 0,6) 0,0805	1,0 (-5,4, 7,5) 0,7546	1,8 (-0,1, 3,7) 0,0675
Não	2930	-1,1 (-3,5, 1,3) 0,3891	-7,0 (-13,1, -0,8) 0,0265	-6,0 (-15,1, 3,1) 0,1957	-0,3 (-2,4, 1,8) 0,7948
Não sabe	1				
**PELO MENOS 12 BEBIDAS ALCOÓLICAS EM 1 ANO**					
Sim	3434	-0,4 (-2,2, 1,3) 0,6268	-4,7 (-9,7, 0,3) 0,0677	-1,4 (-7,9, 5,0) 0,6600	0,9 (-0,9, 2,7) 0,3292
Não	1303	-0,5 (-3,2, 2,1) 0,6935	-5,5 (-12,8, 1,8) 0,1398	-4,9 (-15,9, 6,1) 0,3797	1,8 (-0,4, 4,0) 0,1138
Não sabe	2				
Ausente	488	-0,5 (-2,0, 1,0) 0,5035	-7,1 (-22,3, 8,1) 0,3581	-5,8 (-29,3, 17,7) 0,6281	1,7 (-3,3, 6,6) 0,5120
**MET**					
<4	1355	-0,6 (-2,8, 1,7) 0,6099	-6,7 (-12,9, -0,4) 0,0362	-4,8 (-12,8, 3,2) 0,2381	1,6 (-0,8, 4,0) 0,1847
4-8	1462	-0,5 (-1,5, 0,4) 0,2511	-3,6 (-10,3, 3,0) 0,2818	2,0 (-6,4, 10,5) 0,6361	-0,1 (-2,4, 2,2) 0,9365
≥8	2410	-0,0 (-3,1, 3,1) 0,9958	-1,3 (-9,3, 6,6) 0,7388	-0,7 (-11,9, 10,5) 0,8991	1,8 (-0,8, 4,4) 0,1730

Portanto, a concentração de xantina foi significativamente menor na maioria dos grupos do que no grupo controle, e essa diferença foi mais proeminente em subgrupos de mulheres, raça e algumas características de saúde (como hipertensão e tabagismo, índice de IMC e escore MET). A diferença de concentração de teobromina não atingiu significância estatística em todos os subgrupos (p > 0,05). A diferença na concentração de teobromina entre o grupo com doença cardíaca e o grupo controle não foi estatisticamente significativa e pode ter uma relação fraca com a doença cardíaca. A cafeína mostrou diferenças em alguns subgrupos, no gênero, a diferença para homens foi de 2,2 (p = 0,0081), enquanto não houve diferença significativa para mulheres (p = 0,7220), na raça mexicano-americano houve uma diferença de 9, p = 0,0006, não houve diferença significativa em outros grupos. Para pacientes sem diabetes, a diferença foi de 2,1, p = 0,0165. Portanto, a diferença na concentração de cafeína entre homens e pacientes mexicano-americanos e não diabéticos pode ser mais significativa, mas não há consistência no grupo geral e nos outros grupos.

## Discussão

Com base na análise deste estudo transversal de grande escala de 6 anos, criamos uma Figura Central que resume claramente os principais dados do artigo. A figura mostra que há diferenças significativas nas características basais entre o grupo de controle e o grupo de DCC, particularmente que os indivíduos no grupo DCC são geralmente mais velhos e têm maiores taxas de prevalência de hipertensão, hipercolesterolemia, diabetes, AVC, tabagismo e uso de álcool. A análise dos níveis de cafeína na urina e incidência de DCC revelou uma correlação positiva, especialmente na população mexicana-americana, onde essa associação foi mais pronunciada (razão de chances [OR] = 1,03, intervalo de confiança [IC] de 95%: 1,03–1,05, p = 0,0081). Especificamente, para cada aumento de 1 μmol/L nos níveis de cafeína na urina, o risco de DCC aumentou em 1,03 vezes, com significância estatística (p < 0,01). No entanto, nenhuma associação significativa foi encontrada entre metabólitos urinários de cafeína (como teobromina e teofilina) e DCC. As concentrações de teofilina e hipoxantina foram significativamente menores no grupo DCC em comparação ao grupo controle. A análise de subgrupos mostrou ainda diferenças significativas nos níveis de hipoxantina em diferentes subgrupos de sexo, idade, raça, hipertensão e tabagismo, particularmente em mulheres, pessoas com hipertensão e fumantes. Nenhuma diferença significativa nas concentrações de teobromina e cafeína foi observada na maioria dos subgrupos. Esses resultados sugerem que a relação entre cafeína na urina e DCC pode ser influenciada por fatores como raça e outros fatores fisiológicos e que o metabólito da cafeína hipoxantina pode estar associado à saúde cardiovascular. Este estudo fornece referência valiosa para pesquisa clínica e básica, auxiliando na identificação e intervenção de fatores de risco para DCC.

Estudos anteriores mostraram que o café está positivamente correlacionado com o risco de DCC (OR 1,35, IC 95% 1,05-1,73; p = 0,017) e a ocorrência de hipertensão.^9 , 15^ O consumo moderado ou excessivo de café está associado a um aumento do processo inflamatório, que desempenha um papel crucial no desenvolvimento de DCC.^16^ O consumo excessivo de café, chá e cafeína pode aumentar o risco de mortalidade por todas as causas e morte por doença cardiovascular em pacientes com doença cardiovascular.^17^ O café não filtrado parece aumentar os níveis séricos de triglicerídeos, colesterol e lipoproteína de baixa densidade (LDL).^18 , 19^

No entanto, alguns estudos também mostraram que beber café é seguro e não aumenta o risco de morte. O impacto de diferentes preparações de café nos resultados cardiovasculares e nas taxas de sobrevivência varia. Café descafeinado, pó de café e café instantâneo estão associados a uma redução significativa na incidência e mortalidade de doenças cardiovasculares.^20^ Moer e café instantâneo sem descafeinação estão associados a um menor risco de arritmias cardíacas. Isso sugere que a diversidade de fontes e ingestão de cafeína pode afetar os resultados da pesquisa.^21^

Concluindo, nosso estudo usou cafeína e seus metabólitos na urina como indicadores da ingestão real de café e descobriu que o nível de cafeína na urina estava positivamente correlacionado com a incidência de DCC (OR=1,54, IC 95%: (1,04, 2,3), p = 0,0318), apoiando a confiabilidade do nosso método de pesquisa. Além disso, descobrimos que o nível de paraxantina estava negativamente correlacionado com a incidência de DCC. O risco de morte para aqueles que bebiam pequenas ou grandes quantidades de café foi reduzido em comparação com aqueles que não bebiam café. Aqueles que bebiam uma pequena quantidade de café (1-2 xícaras/dia) e uma grande quantidade de café (>2 xícaras/dia) tinham um risco menor de morte, com os grandes bebedores de café se beneficiando mais.

Vários fatores podem explicar esses resultados. Primeiro, o café é um composto complexo que contém mais de 100 ingredientes bioativos, sendo a cafeína o mais conhecido.^22 , 23^ A ingestão aguda de cafeína pode ativar o sistema nervoso simpático inibindo a fosfodiesterase, aumentando o cálcio citoplasmático e liberando norepinefrina/adrenalina, o que pode aumentar a reserva cardiovascular e manter o fluxo sanguíneo da artéria coronária, reduzindo assim a ocorrência de DCC. Segundo, o efeito antagônico da cafeína na adenosina pode enfraquecer o aumento esperado no fluxo sanguíneo do miocárdio durante o exercício.^24^ Terceiro, descobriu-se que a ingestão de cafeína regula os fatores de risco para DCC, especialmente em não fumantes. Beber café pode ter um efeito potencialmente benéfico na prevenção da calcificação da artéria coronária.^25^ Quarto, o efeito antioxidante da cafeína pode reduzir os danos às células endoteliais e diminuir o risco de DCC. Quinto, os compostos fenólicos no café, especialmente o ácido hidroxicinâmico, têm um efeito protetor no sistema cardiovascular, principalmente alcançado por meio de propriedades anti-inflamatórias e atividade antioxidante. Em contraste, o consumo de curto prazo de café não filtrado pode aumentar os níveis séricos de triglicerídeos, colesterol e LDL.^18^

Neste estudo transversal em larga escala, observamos uma correlação negativa entre os níveis urinários de cafeína e a incidência de DCC em mexicano-americanos, mas nenhuma associação semelhante foi encontrada em outros grupos étnicos. Essa diferença pode estar relacionada às taxas variáveis de incidência de DCC entre diferentes grupos raciais.^26^ Além disso, a variação genética também pode influenciar as respostas individuais à cafeína. Por exemplo, o estudo de Happonen et al.^27^ indicou que certos polimorfismos genéticos (como o gene COMT) podem modular os efeitos da cafeína em eventos cardiovasculares. A literatura^28^ aponta que concentrações plasmáticas elevadas de cafeína em homens estão associadas à redução do fluxo sanguíneo miocárdico de estresse e da reserva de fluxo sanguíneo miocárdico, enquanto respostas hemodinâmicas semelhantes não foram observadas em mulheres. No entanto, em nosso estudo, nenhuma diferença relacionada ao sexo foi observada. Portanto, mais estudos controlados randomizados, de grande amostra e de alta qualidade são necessários para incluir fatores genéticos e elucidar melhor os efeitos modulatórios desses polimorfismos genéticos na relação entre cafeína e saúde cardiovascular.

Na análise de regressão, concluímos que as concentrações de teofilina e hipoxantina no grupo de doença cardíaca foram significativamente menores do que aquelas no grupo controle, especialmente após o ajuste, que ainda mostrou significância estatística, sugerindo que esses compostos podem estar associados à doença cardíaca. Não houve diferença significativa nas concentrações de teobromina e cafeína entre os dois grupos, o que pode não estar diretamente relacionado à doença cardíaca. A teofilina é um produto secundário do metabolismo da cafeína. A diminuição em sua concentração pode refletir função metabólica prejudicada; por exemplo, pacientes com doença cardíaca podem apresentar diminuição na função hepática ou capacidade metabólica geral, levando a uma redução na produção de teofilina. Além disso, também pode ser afetado por inflamação ou estresse oxidativo: a doença cardiovascular é frequentemente acompanhada por inflamação sistêmica ou estresse oxidativo, o que pode interferir na via metabólica da teofilina. Os resultados ajustados indicam um aumento significativo na concentração de teofilina, sugerindo que outros fatores de confusão, como idade, sexo, IMC, etc., podem mascarar a verdadeira associação entre teofilina e doença cardíaca no modelo não ajustado. A diminuição significativa na concentração de teofilina sugere seu valor diagnóstico potencial. No futuro, dados genômicos (como o gene CYP1A2) podem ser combinados para verificar ainda mais se as alterações na concentração de teofilina podem prever o risco de doença cardíaca ou servir como indicadores de monitoramento de eficácia na terapia cardiovascular. Em todos os modelos, os níveis de xantina foram significativamente menores no grupo com doença cardíaca em comparação ao grupo controle (faixa de diferença: -4,1 a -5,1, p < 0,05). A diferença significativa nos níveis de xantina antes e depois do ajuste é consistente, indicando uma associação mais direta com doença cardíaca e menos suscetibilidade a fatores de confusão e sugerindo seu potencial como um marcador metabólico independente para doença cardíaca. Não houve diferença significativa nas concentrações de teobromina e cafeína entre o grupo com doença cardíaca e o grupo controle, que podem não participar diretamente dos processos cardiovasculares e metabólicos, ou suas diferenças podem ser mascaradas por hábitos alimentares e de estilo de vida. No futuro, a paraxantina pode ser incluída na biblioteca de biomarcadores de triagem precoce para doenças cardíacas, e o diagnóstico personalizado pode ser realizado pela combinação de dados genômicos. Desenvolver estratégias de prevenção e controle direcionadas com base nos efeitos regulatórios da teofilina e da hipoxantina, levando em consideração gênero, raça e status metabólico.

A análise de subgrupos mostrou que a paraxantina foi o único composto significativamente reduzido na maioria dos grupos, especialmente em subgrupos como mulheres, pacientes hipertensos, IMC alto e populações com escore MET baixa, sugerindo que pode ser um biomarcador chave potencial para doenças cardíacas. A paraxantina é o principal produto do metabolismo da cafeína e tem certos efeitos anti-inflamatórios e reguladores do metabolismo lipídico. A diminuição significativa na concentração de paraxantina em pacientes com doenças cardíacas pode refletir as características de fatores inflamatórios elevados ou vias metabólicas bloqueadas em doenças cardiovasculares. Em pacientes com hipertensão e IMC alto, há um nível mais alto de estresse metabólico, e a redução da paraxantina pode estar relacionada a distúrbios do metabolismo lipídico, função hepática enfraquecida e estado inflamatório sistêmico. A redução significativa da paraxantina em mulheres, pacientes hipertensos e pacientes com comprometimento funcional grave sugere possíveis diferenças de gênero e o efeito exacerbador da hipertensão nas características metabólicas das doenças cardíacas. A significância da raça branca não hispânica pode refletir diferenças na atividade enzimática metabólica, hábitos de vida e suscetibilidade a doenças entre diferentes raças. Em pesquisas futuras, planejamos conduzir estudos de coorte em larga escala para validar ainda mais a eficácia da xantina como um biomarcador para doenças cardíacas. Não houve diferença estatisticamente significativa na concentração de teofilina e teobromina, indicando uma fraca associação com doenças cardíacas. A teofilina é principalmente um produto intermediário do metabolismo da cafeína, e seu metabolismo é muito influenciado por genes e atividade enzimática hepática (como o polimorfismo do gene CYP1A2). Devido ao fato de que esses processos metabólicos não participam diretamente de alterações fisiopatológicas cardiovasculares, suas alterações de concentração podem não ter uma resposta significativa à doença cardíaca. Além disso, a teofilina tem uma meia-vida mais longa no corpo humano, o que pode mascarar o impacto das flutuações transitórias de concentração na doença cardíaca. Os alcaloides do cacau são um metabólito secundário da cafeína, com baixa eficiência metabólica e concentrações relativamente estáveis no corpo humano, o que pode não refletir significativamente as alterações metabólicas relacionadas à doença cardíaca. As diferenças de concentração de cafeína são significativas apenas em alguns subgrupos específicos, e a tendência geral é inconsistente, o que pode refletir uma correlação fraca com doença cardíaca ou interferência de outros fatores. O aumento significativo na concentração de cafeína em homens pode estar relacionado ao estilo de vida (como hábitos de consumo de café) e maiores taxas metabólicas em homens, enquanto os níveis de estrogênio em mulheres podem interferir no metabolismo da cafeína, reduzindo assim as diferenças. A concentração significativamente elevada em mexicano-americanos pode estar associada a polimorfismos específicos de raça em genes de enzimas metabolizadoras de cafeína, bem como influências culturais e alimentares. A concentração de cafeína em pacientes sem diabetes aumentou significativamente, sugerindo que o diabetes pode afetar a taxa de depuração da cafeína por meio de distúrbios metabólicos. Portanto, uma diminuição significativa na xantina pode se tornar um marcador metabólico para doenças cardíacas, o que pode auxiliar na triagem e intervenção precoces. A descoberta de subgrupos específicos pode ajudar a desenvolver estratégias personalizadas para prevenir e controlar doenças cardíacas.

Nossa pesquisa tem as seguintes vantagens. Primeiro, o estudo usou dados do NHANES para análise de amostra grande, reduzindo efetivamente o viés causado pelo tamanho insuficiente da amostra. Segundo, estratificamos os dados por gênero, idade e raça e consideramos fatores de confusão multivariáveis, como hipertensão e IMC, para análise de regressão multivariável. Esse método nos permite explorar com mais precisão a relação entre cafeína e metabólitos de cafeína na urina e a incidência de DCC. Terceiro, usamos os níveis de cafeína e metabólitos de cafeína na urina como variáveis de exposição para evitar os efeitos do viés de memória, classificação incorreta e diferenças nos tipos de alimentos, em vez de depender da ingestão de cafeína relatada em questionários. Quarto, ao construir diferentes modelos e analisar covariáveis multivariáveis, obtivemos resultados mais confiáveis sobre a relação entre cafeína e seus metabólitos e DCC.

No entanto, este estudo também tem limitações. Primeiro, como este é um estudo transversal, não é possível determinar a relação causal entre metabólitos de cafeína e DCC. Segundo, os sujeitos do estudo vêm principalmente dos Estados Unidos e podem não representar a população global. Terceiro, devido ao método de estratificação dos dados do NHANES e à baixa taxa de incidência de DCC, o tamanho da amostra do grupo de DCC é pequeno, o que pode levar a algum viés nos resultados. Portanto, mais estudos longitudinais e pesquisas multirregionais e multicêntricas são necessários para validar essas descobertas e explorar a relação exata entre cafeína e seus metabólitos e DCC.

## Conclusão

Neste estudo, descobrimos que os níveis de paraxantina urinária estavam inversamente associados ao risco de DCC. Enquanto isso, os níveis de cafeína na urina estavam positivamente associados às taxas de DCC em mexicano-americanos, mas essa associação não foi encontrada em outros grupos. No geral, os níveis de cafeína na urina estavam positivamente associados à incidência de DCC, mas não houve associação significativa em análises estratificadas por sexo. Os resultados deste estudo podem fornecer uma referência para predição e diagnóstico clínico e são de grande importância na prática clínica, mas mais estudos prospectivos em larga escala são necessários para verificar essas conclusões.

## *Material suplementar

Para informação adicional, por favor, clique aqui


