# Supplementary figures and images for: Estudo sobre os Níveis Urinários de Cafeína e seus Metabólitos em Adultos Americanos com Doença Cardíaca Coronária: Estudo Transversal NHANES (2009-2014)
Source: Arq Bras Cardiol. 2025 Jun 4;122(6):e20240425. [Article in Portuguese] doi: 10.36660/abc.20240425 (PMC12217927; doi:10.36660/abc.20240425)

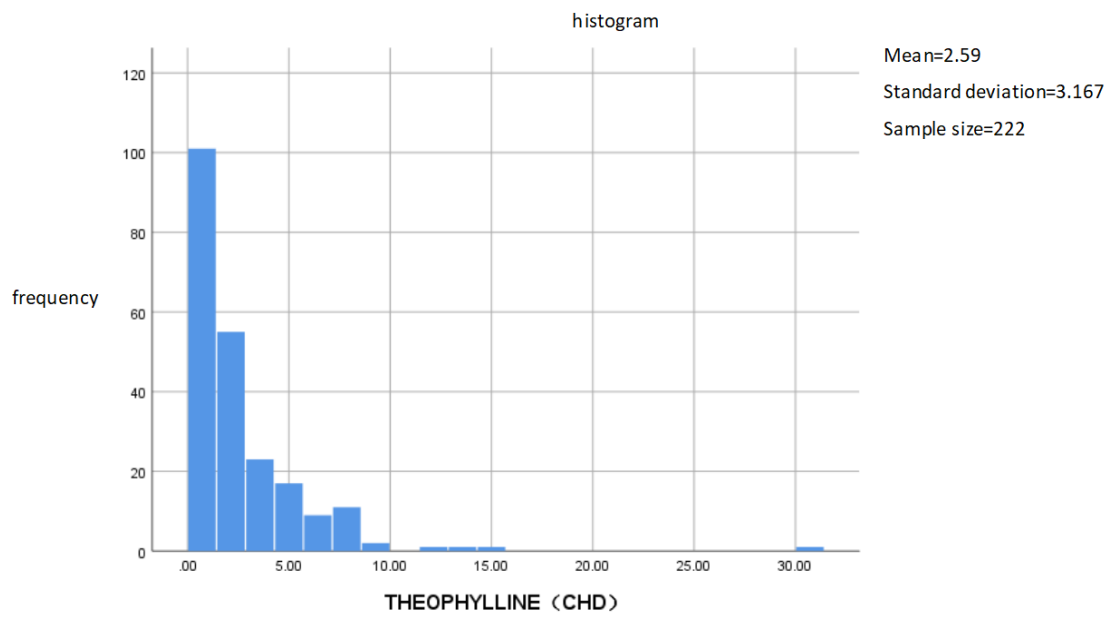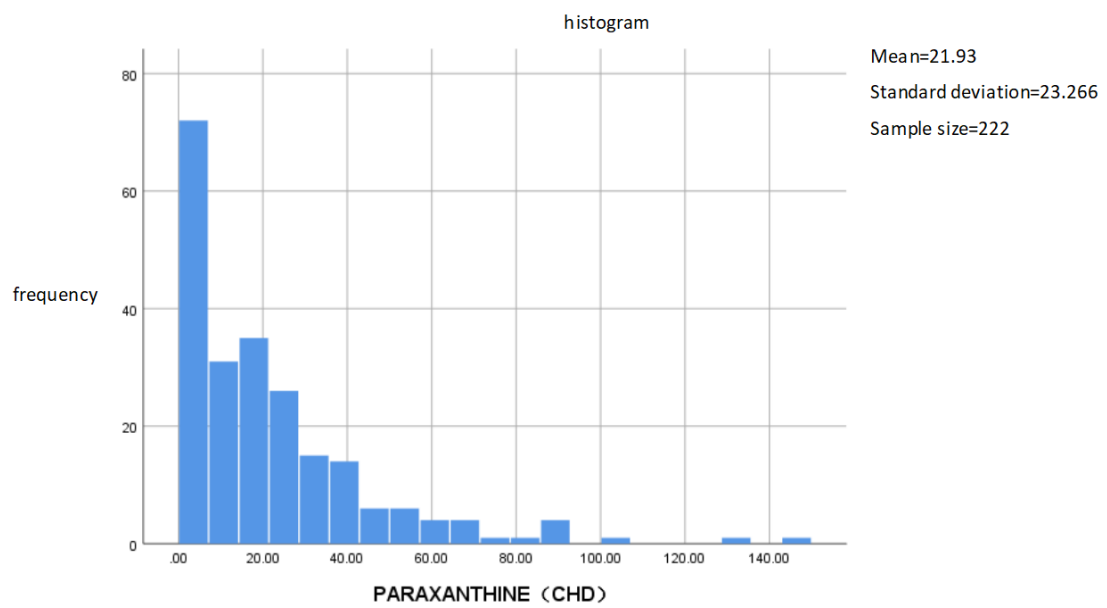

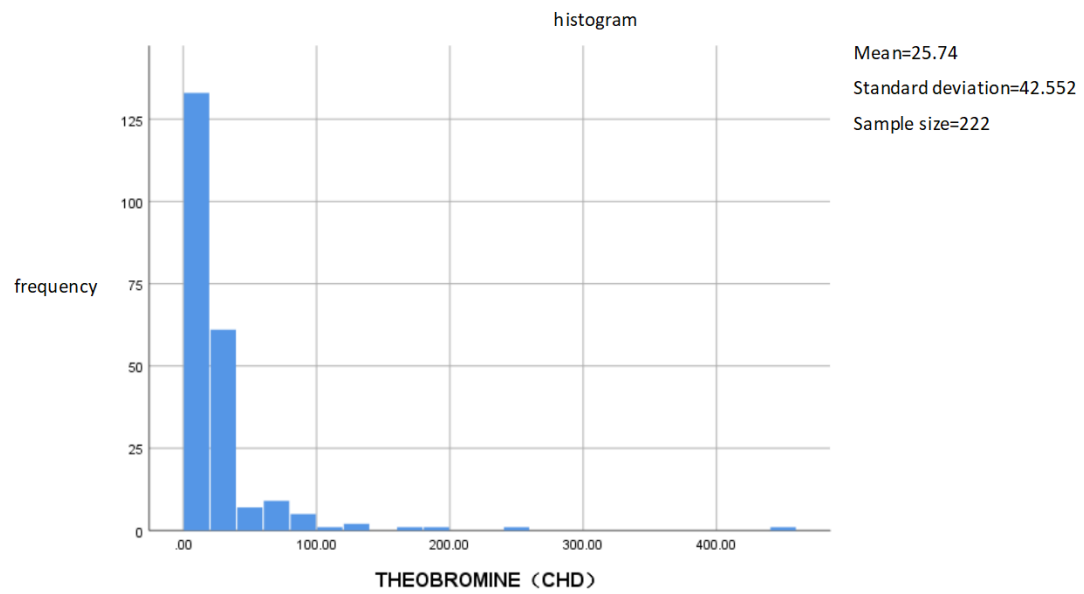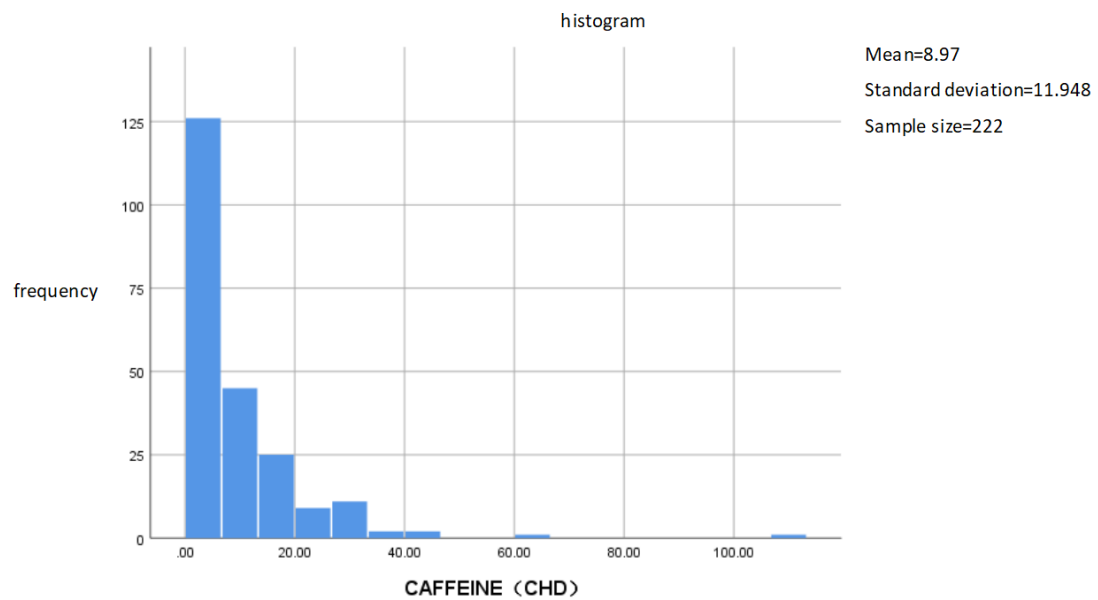

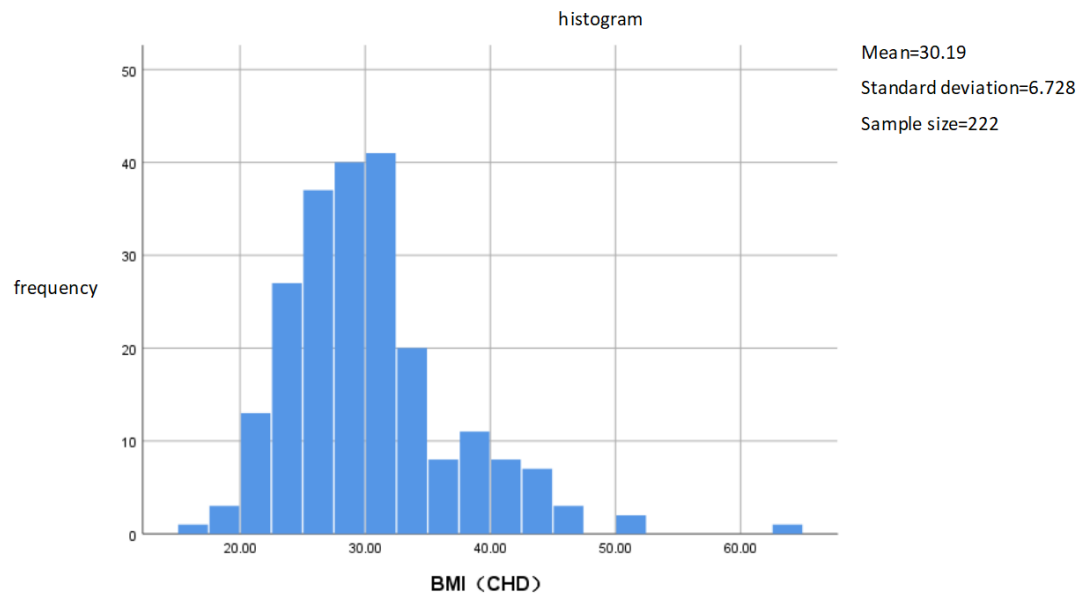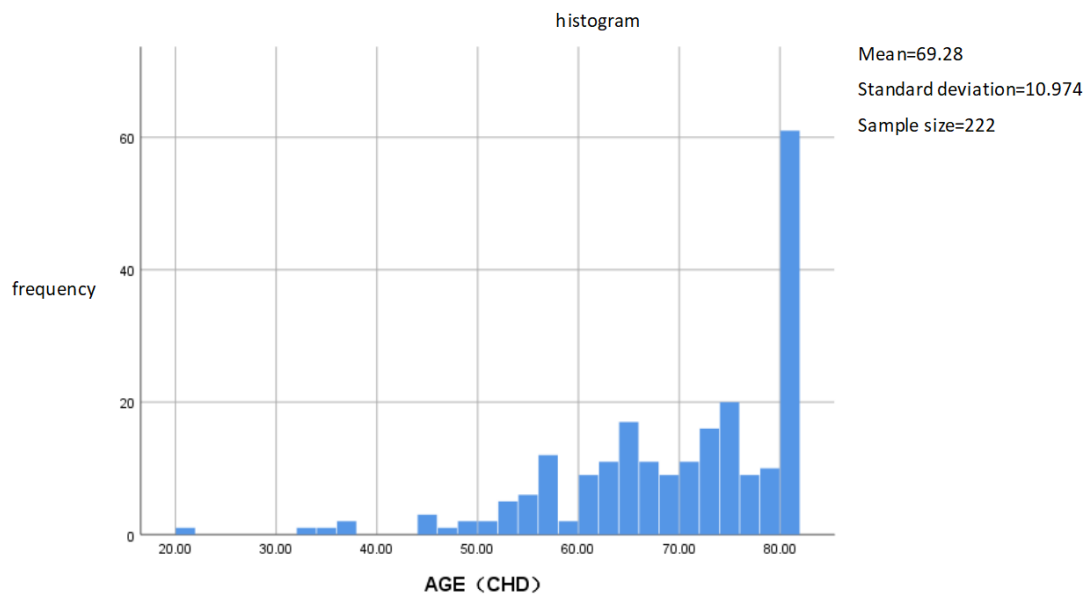

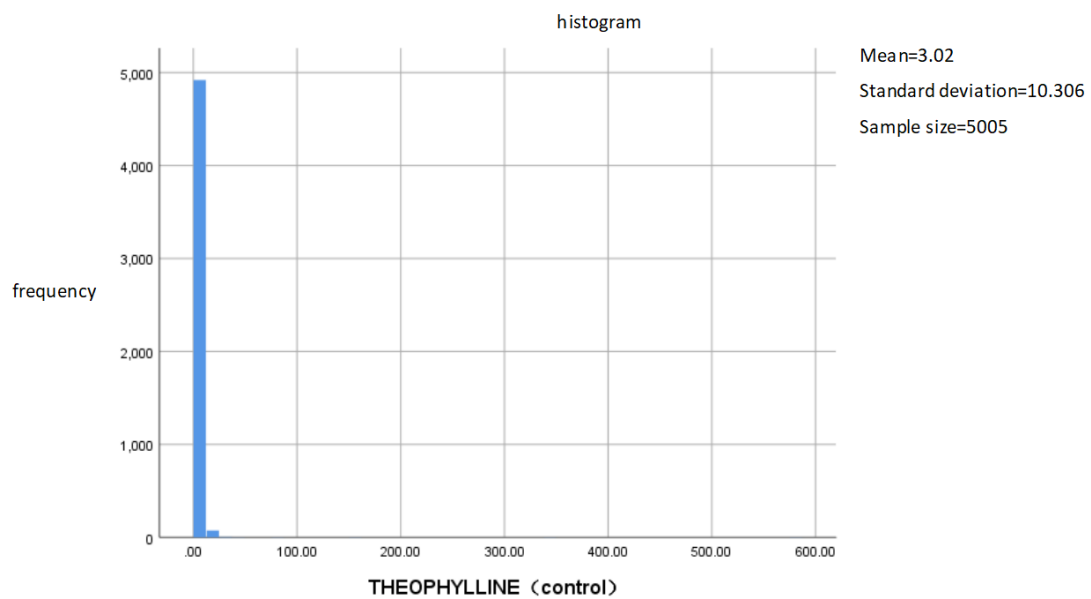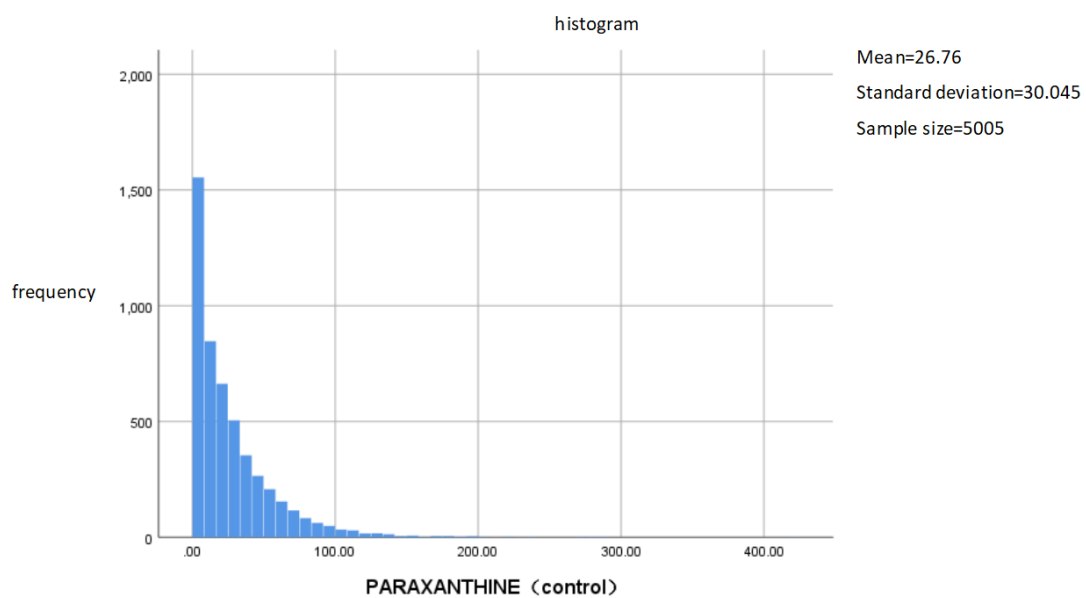

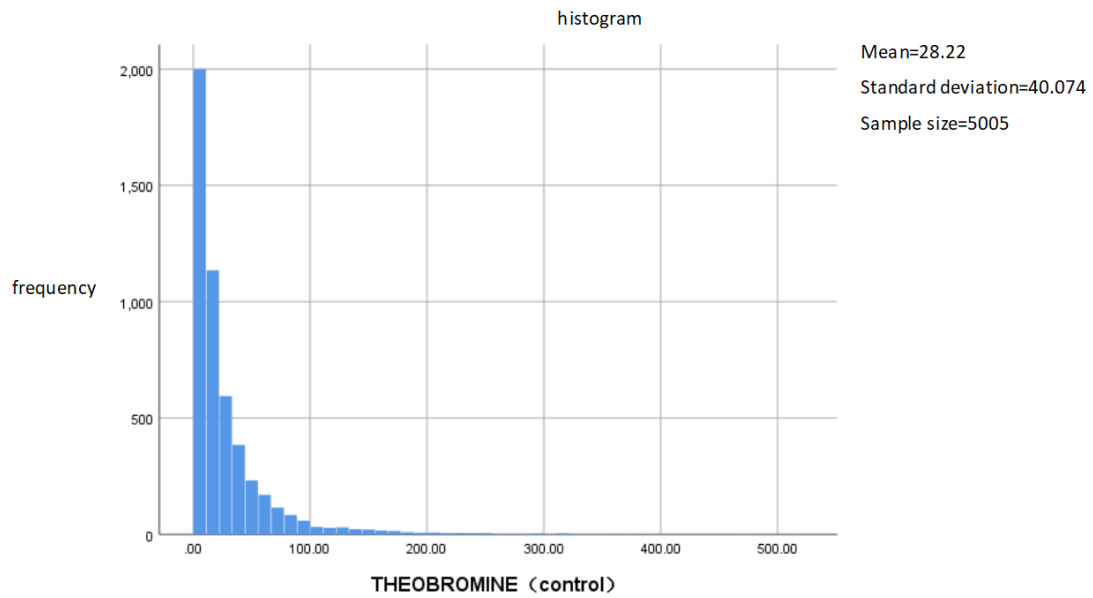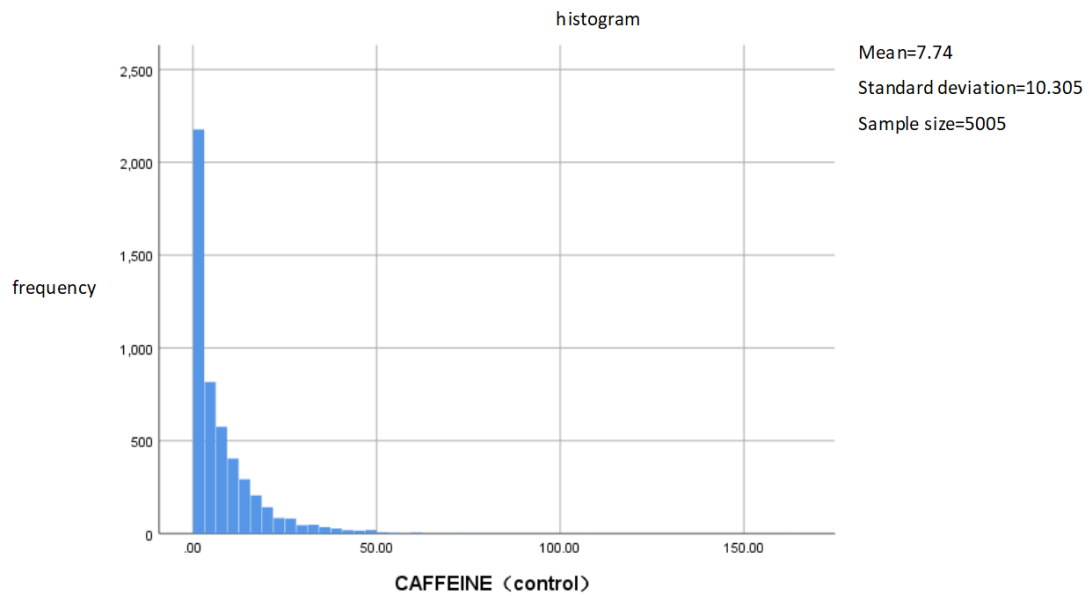

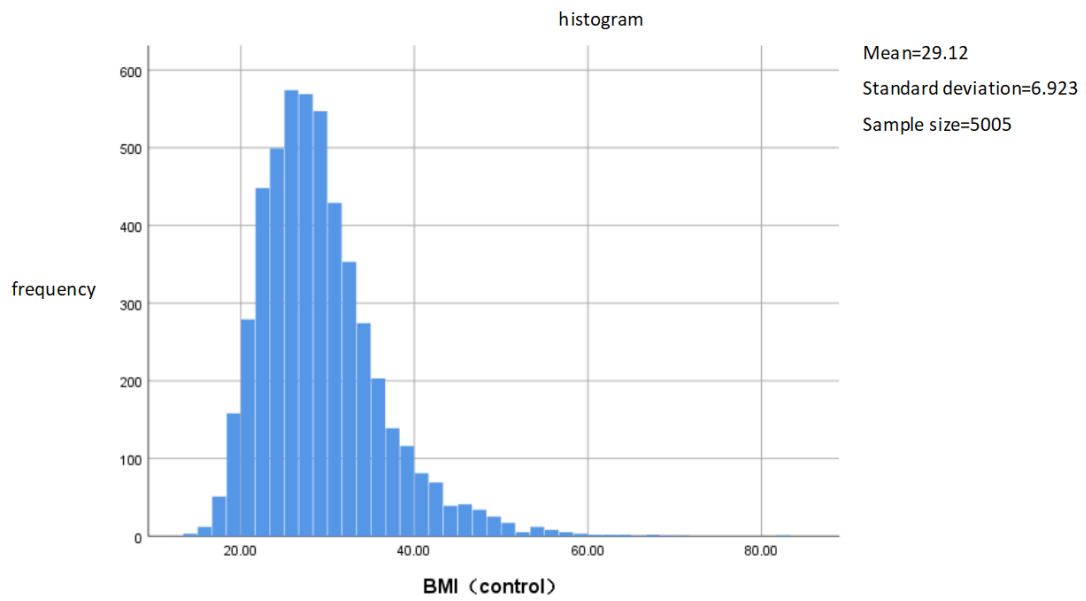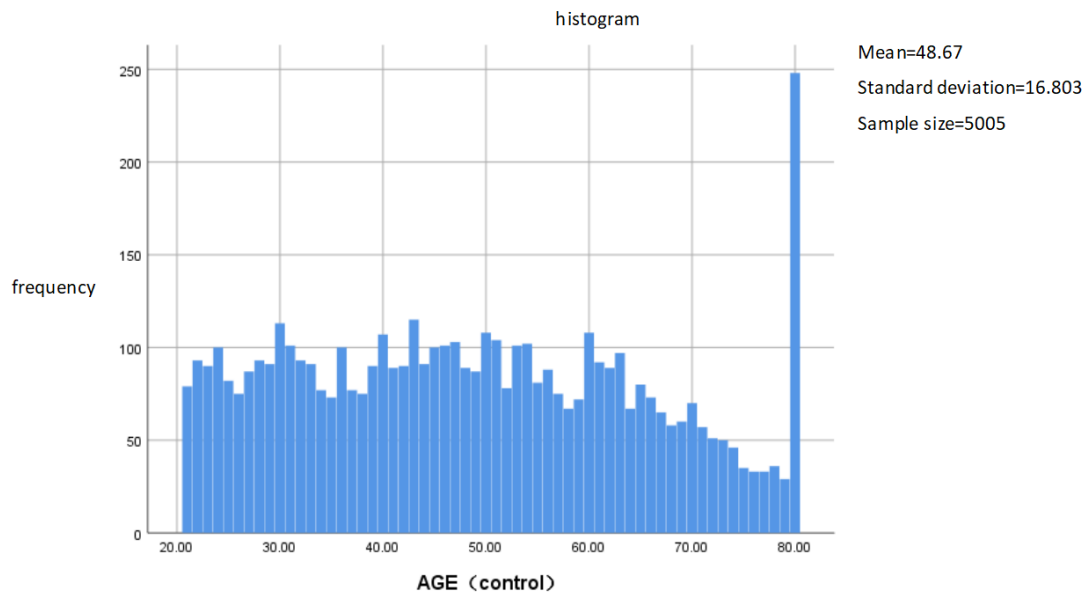

Supplement: Supplementary file 1 [file 2024-0425_appendix.pdf]
