# Supplementary material for: Estudo sobre os Níveis Urinários de Cafeína e seus Metabólitos em Adultos Americanos com Doença Cardíaca Coronária: Estudo Transversal NHANES (2009-2014)
Source: Arq Bras Cardiol. 2025 Jun 4;122(6):e20240425. [Article in Portuguese] doi: 10.36660/abc.20240425 (PMC12217927; doi:10.36660/abc.20240425)
Supplement: Translation

# Translation: Study on Urinary Caffeine and Caffeine Metabolites Levels in American Adults with Coronary Heart Disease: NHANES Cross-Sectional Study (2009-2014)

**DOI:** 10.36660/abc.20240425i

**Authors:** Qianqian Li, Gan Ke, Mengdie Liu, Yanjun Chen, Ye Luo, Liangxian Qiu

**Affiliations:** 1 China Shenzhen University, Shenzhen – China; 2 China Peking University Shenzhen Hospital – Department of Cardiovascular Medicine, Shenzhen – China

**Keywords:** Caffeine, Cardiovascular Diseases, Racial Groups

## Abstract

**Background:**

In recent years, the impact of diet and lifestyle on coronary heart disease (CHD) has received widespread attention. Caffeine, a common dietary component, and its metabolites may be related to cardiovascular health.

**Objective:**

The study aims to investigate the association between urinary levels of caffeine and its metabolites and the incidence of CHD and to assess the relationship between urinary caffeine levels and CHD incidence in different populations.

**Methods:**

Data were analyzed using multivariable logistic regression models and smooth curve fitting methods. Specific steps included extracting relevant data from the NHANES database for 2009 to 2014; categorizing participants into a control group (n = 5005) and a CHD group (n = 222) based on their CHD status; measuring and recording urinary levels of caffeine and its metabolites; applying multivariable logistic regression models to evaluate the association between urinary caffeine and its metabolites levels and CHD incidence; and using smooth curve fitting methods to explore the nonlinear relationships of these associations further. The significance level was set at 5% in the statistical analysis to determine the statistical significance of the results.

**Results:**

Urinary paraxanthine levels were inversely related to CHD risk. Urinary caffeine levels were positively associated with CHD incidence in Mexican Americans, but this association was not observed in other populations.

**Conclusion:**

Lower urinary paraxanthine levels may indicate a reduced risk of CHD, while higher urinary caffeine levels may be associated with increased CHD risk in specific populations.

## Research background and significance

In recent years, coronary heart disease (CHD) has become one of the leading causes of death, accounting for over 370,000 deaths annually.^1^Alongside traditional risk factors such as smoking, hyperlipidemia, hypertension, diabetes, and obesity, new risk factors have been identified, including systemic inflammation, gestational diabetes, preeclampsia, and early or surgical menopause.

Dietary modifications have been shown to reduce the incidence of CHD,^2^with natural pharmacological compounds, especially caffeine, often used as complementary therapies for cardiovascular diseases.^3^Coffee, the most widely consumed beverage globally, contains caffeine (1,3,7-trimethylxanthine), which is ingested through food and beverages.^4 , 5^Although caffeine may cause a temporary increase in blood pressure in non-habitual drinkers, long-term coffee consumption can help prevent arrhythmias and heart failure and improve insulin resistance, thus lowering diabetes risk.

Studies have shown that coffee consumption is inversely related to all-cause mortality, and habitual coffee drinking is associated with reduced risks of cardiovascular diseases and adverse cardiovascular outcomes, including CHD, congestive heart failure, and stroke. Long-term coffee drinking is also associated with lower risks of type 2 diabetes, depression, obesity, and cancer.^6 , 7^Specifically, increased levels of caffeine and theophylline in urine are negatively correlated with cardiovascular diseases,^8^with this association being more pronounced in women.^9^However, most current research is based on surveys of coffee consumption habits, and different brands, origins, and types of coffee contain varying amounts of caffeine. Few studies have examined the relationship between caffeine metabolites and cardiovascular diseases, and existing research often overlooks differences caused by coffee type, origin, and production processes, with potential errors in questionnaire surveys impacting the results.

A reliable indicator of coffee intake is the amount of caffeine, and its metabolites excreted in the urine. Individual differences in caffeine metabolism may lead to variations in circulating caffeine and metabolite concentrations. To better understand the association between urinary caffeine and its metabolites and CHD, we conducted a cross-sectional study using data from the 2009-2014 National Health and Nutrition Examination Survey (NHANES) to explore potential relationships between caffeine, its metabolites, and CHD incidence.

## Materials and Methods

### Study population

NHANES is a cross-sectional nutrition survey in the United States that uses a multistage stratified probability sampling method and has been approved by the National Center for Health Statistics to collect and disseminate data.^10^This study selected relevant data from adults (age ≥ 20 years old) who participated in NHANES from 2009 to 2014 for inclusion in the study.^11^The authors did not have access to information that could identify individual participants during or after data collection.

Data from a total of 30,468 participants between 2009 and 2014 were initially included in this study. Participants in this study were younger than 20 years old (n = 13,261), had incomplete CHD data (n = 67), and incomplete data on caffeine and caffeine metabolites (n = 11913) were excluded, resulting in 5227 subjects included ( Figure 1 ).

**Figure 1:** – Participant selection flowchart.

### Exposure factors

Since theobromine (3,7-dimethylxanthine), theophylline (1,3-dimethylxanthine), and paraxanthine (1,7-dimethylxanthine) are the main caffeine metabolizers in human substances,^12^therefore in this study, urinary levels of caffeine and caffeine metabolites were used as exposure variables. Urine samples were collected between 22 and 24 a.m. after participants in the study underwent a 9-hour overnight fast. Urine samples were then received, stored, and shipped to the Centers for Disease Control and Prevention Laboratory Sciences Division at the National Environmental Center in Atlanta, Georgia, to check them. Finally, high-performance liquid chromatography-electrospray ionization-tandem quadrupole mass spectrometry (HPLC-ESI-MS/MS) and stable isotope-labeled internal standards were used to determine caffeine and its metabolites in urine,^13^and Data were reviewed. The performing laboratory receives uncertain values or incomplete data for verification.

### Outcome variable

CHD diagnosis is based on the patient’s clinical records and imaging examination results (such as coronary angiography) and is collected by experienced interviewers using computer-aided personal interview (CAPI) technology. The questionnaire is available on the NHANES website.

### Covariates

This study included covariates that may influence the relationship between caffeine and caffeine metabolites and CHD, including gender, age, race, history of high blood pressure, history of high cholesterol, history of diabetes, history of stroke, and smoking for at least 100 years in a lifetime. Data such as expenditure (%), drinking at least 12 alcoholic drinks/year, MET(Physical activity status), and body mass index (BMI) (kg/m^2^).^11^Among them, BMI is calculated by weight/height squared. Qualified health technicians at mobile examination centers collected body measurements. Other covariates were collected as outcome variables through the CAPI system.

### Statistical analysis

We used R software and Empower Statistics for statistical analysis.^14^When the p-value of the analysis result < 0.05, the result was considered significant. Missing continuous variables among included subjects were excluded while missing categorical variables formed a separate group. The normality test for all continuous variables is conducted using the Kolmogorov-Smirnov test, combined with histogram visualization for analysis. If the data follows a normal distribution, it is represented by the mean ± standard deviation, and independent sample t-tests are used for group comparisons. If the data does not follow a normal distribution, it is represented by the median and interquartile range (Q1-Q3), and the Mann-Whitney U test is used for group comparisons. Following the normality test, we found that the continuous data do not adhere to a normal distribution (see Appendix 1 for details). For continuous variables with a non-normal distribution, the data are summarized using the median and interquartile range (Q1-Q3), and group comparisons are conducted using the Mann-Whitney U test. Categorical data were described as absolute (n) and relative (%) frequencies, and the Chi-Square test was performed to compare differences between groups. When the expected frequency is less than 10, Fisher’s exact test is used. Multivariable logistic regression analysis evaluated the relationship between urinary caffeine and caffeine metabolite levels and CHD. For continuous data with non-normal distribution, we use the quartile grouping method (Q1, Q2, Q3, Q4) to divide the data into four parts, providing a better representation of its distribution. Q4 represents the quarter of the data, not the fifth segment of a quintile analysis. In our study, the dataset is divided into four quartiles (Q1, Q2, Q3, Q4) based on the values of the variable of interest. Q1 represents the lowest 25% of the data, Q2 represents the middle 50%, and Q3 represents the upper 25%. Q4, therefore, represents the final quartile, which includes the top 25% of the data, extending from Q3 to the maximum value. It is important to note that Q4 is not an additional boundary point but the uppermost range of the data, covering the final 25%. By using the Q1-Q4 quartile grouping, we are able to clearly demonstrate the trend of the variable of interest across different data distribution intervals. Specifically, Q1 represents the lowest 25% of the data, providing a baseline for comparisons with the higher quartiles (Q2, Q3, Q4). Compared to the Q2, Q3, and Q4 groups, we can more clearly observe the differences between different distribution levels. Q4, representing the top 25% of the data, allows us to highlight the characteristics of the high-value group and its differences from other groups. This grouping method helps better reveal trends and changes in the data distribution, offering a more detailed analytical perspective. We chose Q1 (the lowest 25%) as the “Reference” group because it represents the low-risk/low-level population, making it suitable as a control group. In this grouping, the other groups (Q2, Q3, Q4) represent progressively higher levels, which helps clearly demonstrate the differences between the lower-level and higher-level groups.

This study created a total of 3 related models, first creating a model 1 without adjusting variables, then creating an adjusted model 2 using variables including gender, age, and race or ethnicity, and finally creating an adjusted model 2 by adjusting gender, age, race, height Model 3 was obtained based on the history of blood pressure, high cholesterol, diabetes, stroke, smoking at least 100 cigarettes in life (%), drinking at least 12 alcoholic drinks/year, and BMI (kg/m^2^). Analysis of various covariates based on the above three models further clarified the relationship between urinary caffeine and its metabolites and CHD. Smooth curve fitting was performed to further investigate the relationship between caffeine and caffeine metabolite levels and CHD after controlling for some variables. In addition, we also conducted subgroup analysis to explore the associations between variables further and identify the specific characteristics of different subgroups by segmenting the population.

### Ethical approval

The data for this study came from the NHANES website. Therefore, it does not require institutional review board (or ethics committee) approval.

## Results

### Basic characteristics of the research population

The normality test revealed that the continuous data did not follow a normal distribution (see Appendix 1). Non-normally distributed continuous variables are presented as a median and interquartile range, with group differences assessed using the Mann-Whitney U test. Categorical variables are expressed as N (%), and group differences are analyzed using the chi-square or Fisher’s exact test when expected frequencies are below 10. A p-value of <0.05 indicates statistical significance.

The participants in this study were divided into a control group (n = 5005) and a CHD group (n = 222). The analysis revealed significant differences in baseline characteristics between the two groups, with the exception of BMI, urinary caffeine, and caffeine metabolites—for example, age, gender, race, MET score, etc. Notably, individuals in the CHD group were significantly older (72 (IQR: 63-80)years vs. 48 (IQR:34-62)years, p < 0.001) and exhibited a higher prevalence of hypertension, hypercholesterolemia, diabetes, stroke, as well as smoking and drinking histories (all p < 0.001). Additionally, there were observable differences in the ethnic distribution between the two groups (p = 0.001),

There was a significant difference in Paraxanthine levels between the two groups (p = 0.018), as summarized in Table 1 .

**Table 1:** – Basic characteristics and levels of caffeine and caffeine metabolites in the urine of study participants

Variables	Heart disease group (n=222)	Control group (n=5005)	Standardized diff.	p-value	p-value*
**AGE (year)**	72(63-80)	48(34-62)	1.5 (1.3; 1.6)	<0.001	
**SEX**			0.4 (0.3; 0.6)	<0.001	-
Male	153 (68.9%)	2390 (47.8%)			
Female	69 (31.1%)	2615 (52.2%)			
**RACE**			0.5 (0.4; 0.6)	<0.001	-
Mexican American	17 (7.7%)	700 (14.0%)			
Other Hispanic	18 (8.1%)	504 (10.1%)			
Non-Hispanic White	146 (65.8%)	2111 (42.2%)			
Non-Hispanic Black	26 (11.7%)	1074 (21.5%)			
Other Race - Including Multi-Racial	15 (6.8%)	616 (12.3%)			
**HIGH BLOOD PRESSURE**			1.0 (0.8; 1.1)	<0.001	
Yes	173 (77.9%)	1735 (34.7%)			
No	48 (21.6%)	3262 (65.2%)			
Do not know	1 (0.5%)	8 (0.2%)			
**HIGH CHOLESTEROL LEVEL**			1.0 (0.9; 1.1)	<0.001	-
Yes	163 (73.4%)	1552 (31.0%)			
No	56 (25.2%)	2887 (57.7%)			
Do not know	3 (1.4%)	31 (0.6%)			
Missing	0 (0.0%)	535 (10.7%)			
**DIABETES**			0.5 (0.4; 0.6)	<0.001	-
Yes	65 (29.3%)	550 (11.0%)			
No	147 (66.2%)	4333 (86.6%)			
Borderline	10 (4.5%)	117 (2.3%)			
Do not know	0 (0.0%)	5 (0.1%)			
**STROKE**			0.4 (0.3; 0.5)	<0.001	
Yes	29 (13.1%)	138 (2.8%)			
No	192 (86.5%)	4864 (97.2%)			
Do not know	1 (0.5%)	3 (0.1%)			
**SMOKED AT LEAST 100 CIGARETTES IN LIFE**			0.4 (0.2; 0.5)	<0.001	
Yes	136 (61.3%)	2160 (43.2%)			
No	86 (38.7%)	2844 (56.8%)			
Do not know	0 (0.0%)	1 (0.0%)			
**AT LEAST 12 ALCOHOL DRINKS IN 1 YEAR**			0.2 (0.0; 0.3)	0.195	-
Yes	156 (70.3%)	3278 (65.5%)			
No	54 (24.3%)	1249 (25.0%)			
Do not know	0 (0.0%)	2 (0.0%)			
Missing	12 (5.4%)	476 (9.5%)			
**MET**			0.4 (0.3; 0.6)	<0.001	-
<4	91 (41.0%)	1264 (25.3%)			
4-8	70 (31.5%)	1392 (27.8%)			
≥8	61 (27.5%)	2349 (46.9%)			
**BMI (kg/m** ^**2**^ **)**	28.86 (25.803; 32.975)	28 (24.3.32.42)	0.2 (0.0; 0.3)	0.023	0.007
**THEOPHYLLINE (μmol/L)**	1.645 (0.625; 3.538)	1.88 (0.639.3.84)	0.1 (-0.1; 0.2)	0.532	0.367
**PARAXANTHINE (μmol/L)**	16.6 (4.738; 29.325)	17.5 (5.72; 37.4)	0.2 (0.0; 0.3)	0.018	0.098
**THEOBROMINE (μmol/L)**	13.5 (6.285; 29.8)	15.3 (6.36; 34)	0.1 (-0.1; 0.2)	0.367	0.289
**CAFFEINE (μmol/L)**	5.465 (1.533;12.2)	4.23 (1.01; 10.8)	0.1 (-0.0; 0.2)	0.086	0.045

BMI: body mass index. The normality test revealed that the continuous data did not follow a normal distribution (see Appendix 1). Non-normally distributed continuous variables are presented as a median and interquartile range, with group differences assessed using the Mann-Whitney U test. Categorical variables are expressed as N (%), and group differences are analyzed using the chi-square or Fisher’s exact test when expected frequencies are below 10. A p-value of <0.05 indicates statistical significance.

### The relationship between urinary caffeine levels and coronary heart disease

Results of the relationship between urinary caffeine levels and CHD are shown in Table 2 . First, without adjusting for different covariates, urinary caffeine levels were consistent with CHD without statistical significance (all p > 0.05). The continuous caffeine levels were then converted into categorical variables (quartiles) of the four subgroups, and it can be seen that the urinary caffeine level in the Q4 group was positively associated with the incidence of CHD (odds ratio [OR] = 1.54, 95% Confidence interval [CI]: (1.04, 2.3), p = 0.0318). That is, for every unit (μmol/L) increase in urinary caffeine level, the risk of CHD is 1.54 times higher than before the increase.

**Table 2:** – Relationship between urinary caffeine levels (μmol/L) and coronary heart disease. Model 1: Covariates unadjusted. Model 2: Adjust for age, gender, and race of subjects. Model 3: Adjust for age, gender, race, high blood pressure, high cholesterol, diabetes, stroke, smoking, drinking, and BMI

	Model 1 OR (95% CI) p value	Model 2 OR (95% CI) p value	Model 3 OR (95% CI) p value
**Caffeine (μmol/L)**	1.01 (1.00, 1.02) 0.0859	1.00 (0.99, 1.01) 0.9476	0.99 (0.98, 1.01) 0.3973
**Caffeine categories**			
Q1	Reference	Reference	Reference
Q2	1.37 (0.91, 2.06) 0.1257	1.20 (0.78, 1.85) 0.3998	1.13 (0.73, 1.77) 0.5799
Q3	1.42 (0.95, 2.13) 0.0874	1.18 (0.77, 1.81) 0.4479	1.01 (0.65, 1.59) 0.9487
Q4	1.54 (1.04, 2.30) 0.0318	1.10 (0.72, 1.68) 0.6571	0.94 (0.61, 1.47) 0.8006
**Sex**			
Men	1.0 (1.0, 1.0) 0.009	1.0 (1.0, 1.0) 0.545	1.0 (1.0, 1.0) 0.804
Female	1.0 (1.0, 1.0) 0.721	1.0 (1.0, 1.0) 0.282	1.0 (1.0, 1.0) 0.155
**Race**			
Mexican American	1.03 (1.01, 1.05) 0.0081	1.03 (1.00, 1.05) 0.0700	1.02 (0.98, 1.06) 0.2327
Other Hispanic	1.03 (0.99, 1.07) 0.1409	1.02 (0.97, 1.06) 0.4538	1.00 (0.95, 1.05) 0.9737
Non-Hispanic White	0.99 (0.98, 1.01) 0.3734	0.99 (0.97, 1.01) 0.2670	0.98 (0.97, 1.00) 0.0826
Non-Hispanic Black	1.00 (0.95, 1.05) 0.9936	0.99 (0.94, 1.04) 0.6900	0.99 (0.93, 1.04) 0.6351

Analyzes were then stratified by gender and race. In sex-stratified analyses, urinary caffeine levels were not associated with the incidence of CHD (odds ratio [OR] = 1.0, 95% confidence interval [CI]: (1.0, 1.0), p = 0.009). In an ethnic subgroup analysis, urinary caffeine levels were positively associated with the incidence of CHD in Mexican Americans (odds ratio [OR] = 1.03, 95% confidence interval [CI]: (1.03, 1.05), p = 0.0081 ), that is, for every unit (μmol/L) increase in urinary caffeine level, the risk of CHD increases by 1.03 times. The results of other subgroups showed that no significant relationship was observed between urinary caffeine metabolites and CHD (all p > 0.05). Figure 2A shows a smooth curve fit of the relationship between urinary caffeine levels and the incidence of CHD.

**Figure 2:** – Smooth curve fitting of the relationship between urinary caffeine and caffeine metabolite levels (μmol/L) and the incidence of coronary heart disease. The red line represents the smooth curve fit between caffeine and caffeine metabolites and coronary heart disease. The blue line represents its 95% confidence interval. Adjusted for participant gender, age, race, high blood pressure, high cholesterol, diabetes, stroke, smoking, alcohol consumption, and BMI.

### Association between urinary caffeine metabolite levels and coronary heart disease

The results of the relationship between urinary caffeine metabolite levels and CHD are shown in Table 3 . First, without adjustment for different covariates, urinary theophylline and theobromine levels were consistent with CHD and had no statistical significance (all p > 0.05). Continuous urinary theophylline and theobromine levels were then converted into categorical variables (quartiles). None are statistically significant. After adjusting covariates, there was no statistical significance between urinary theophylline and theobromine levels and CHD (all p > 0.05). A smooth curve fitting of the relationship between urinary theophylline and theobromine levels and the incidence of CHD is shown in Figures 2B and C .

**Table 3:** – Relationship between urinary caffeine metabolite levels (μmol/L) and coronary heart disease. Model 1: Covariates unadjusted. Model 2: Adjust for age, gender, and race of subjects. Model 3: Adjust for age, gender, race, high blood pressure, high cholesterol, diabetes, stroke, smoking, drinking, and BMI

	Model 1 OR (95% CI) P value	Model 2 OR (95% CI) P value	Model 3 OR (95% CI) P value
**Theophylline (μmol/L)**	0.98 (0.93, 1.02) 0.3320	0.98 (0.94, 1.02) 0.3084	0.97 (0.93, 1.02) 0.2592
**Theophylline categories**			
Q1	Reference	Reference	Reference
Q2	1.10 (0.76, 1.59) 0.6243	1.00 (0.67, 1.49) 0.9914	0.94 (0.62, 1.42) 0.7712
Q3	0.94 (0.64, 1.38) 0.7660	0.89 (0.59, 1.34) 0.5788	0.83 (0.54, 1.27) 0.3800
Q4	0.92 (0.63, 1.36) 0.6883	0.90 (0.59, 1.36) 0.6020	0.85 (0.55, 1.31) 0.4527
**Theobromine (μmol/L)**	1.00 (0.99, 1.00) 0.3674	1.00 (1.00, 1.00) 0.6186	1.00 (1.00, 1.00) 0.9285
**Theobromine categories**			
Q1	Reference	Reference	Reference
Q2	1.12 (0.77, 1.62) 0.5605	1.19 (0.80, 1.76) 0.3965	1.09 (0.73, 1.64) 0.6724
Q3	1.09 (0.75, 1.58) 0.6515	1.14 (0.76, 1.70) 0.5217	1.10 (0.73, 1.68) 0.6430
Q4	0.76 (0.51, 1.14) 0.1816	0.84 (0.54, 1.30) 0.4293	0.89 (0.57, 1.40) 0.6291
**Paraxanthine (μmol/L)**	0.99 (0.99, 1.00) 0.0177	1.00 (0.99, 1.00) 0.1037	0.99 (0.99, 1.00) 0.1154
**Paraxanthine categories**			
Q1	Reference	Reference	Reference
Q2	0.90 (0.62, 1.31) 0.5866	0.76 (0.51, 1.13) 0.1789	0.74 (0.49, 1.11) 0.1461
Q3	1.01 (0.71, 1.45) 0.9409	0.99 (0.67, 1.45) 0.9396	0.97 (0.65, 1.45) 0.8707
Q4	0.65 (0.43, 0.97) 0.0360	0.68 (0.44, 1.04) 0.0779	0.65 (0.42, 1.03) 0.0655

The results of model 1 showed that elevated urinary paraxanthine levels were associated with a reduced incidence of CHD (OR = 0.99, 95% CI (0.99, 1.0), p = 0.0177). Models 2 and 3 showed no significant relationship between urinary paraxanthine levels and CHD after adjusting for covariates. For multivariable regression analysis, we further transformed urinary paraxanthine levels from a continuous variable into a categorical variable (four categories). In the group with the highest urinary paraxanthine levels (Q4), urinary paraxanthine levels were inversely associated with reduced incidence of in model 1 (OR = 0.65, 95% CI (0.43, 0.97), p = 0.036), but there was no statistical significance in other models (all p > 0.05).

### Differences in urinary caffeine and caffeine metabolite levels (μ mol/L) between the control group and the coronary heart disease group

Explore the differences in the concentrations of theophylline, paraxanthine, theobromine, and caffeine between the heart disease group and the control group. The study used unadjusted, Adjust I, and Adjust II models for analysis. The table lists four compounds - Theophylline, Paraxanthine, Theobromine, and Caffeine. Divided into the control group, the population without heart disease is set as the control group, with a sample size of 5005, and the heart disease group, the population with heart disease, with a sample size of 222. The comparison of each compound between the heart disease group and the control group is represented by the difference (95% confidence interval) and p-value: the difference is the average concentration difference between the heart disease group and the control group. The 95% confidence interval (in parentheses) represents the estimated range of differences. The p-value is used to test whether the difference is statistically significant (p-value<0.05 is considered statistically significant).

The results shown in Table 4 indicate that Theophylline Non-adjusted had a difference of -0.4 (-0.9, 0.1), p = 0.0923, which did not reach statistical significance. Adjust I: The difference is -0.8 (-1.5, -0.1), p = 0.0196, significant. Adjust II: The difference is -0.7 (-1.5, -0.0), p = 0.0412, significant. After adjustment, the concentration of theophylline in the heart disease group was significantly lower than that in the control group. Paraxanthine Non-adjusted: The difference is -4.8 (-8.0, -1.7), p = 0.0028, significant. Adjust I: The difference is -5.1 (-8.4, -1.8), p = 0.0023, significant. Adjust II: The difference is -4.1 (-7.3, -0.8), p = 0.0135, significant. Paraxanthine levels were significantly lower in all models compared to the control group. Theobromine Non-adjusted: The difference is -2.5 (-8.2, 3.2), p = 0.3925, not significant. Adjust I: The difference is -0.1 (-5.9, 5.7), p = 0.9689, not significant. Adjust II: The difference is -0.1 (-5.9, 5.7), p = 0.9770, not significant. There was no significant difference in the concentration of theobromine between the heart disease group and the control group. Caffeine Non-adjusted: The difference is 1.2 (-0.4, 2.8), p = 0.1327, not significant. Adjust I: The difference is -0.9 (-2.5, 0.8), p = 0.2966, not significant. Adjust II: The difference is -0.5 (-2.1, 1.1), p = 0.5534, not significant. After adjustment, there was no significant difference in caffeine concentration between the two groups. Therefore, we can conclude that the concentrations of theophylline and hypoxanthine in the heart disease group were significantly lower than those in the control group, especially after adjustment, which still showed statistical significance, suggesting that these compounds may be associated with heart disease. There was no significant difference in the concentrations of theobromine and caffeine between the two groups, which may not be directly related to heart disease. The impact of different adjustment models on the results is limited, but the adjusted results are more reliable.

**Table 4:** – Differences in urinary caffeine and caffeine metabolite levels (μ mol/L) between the control group and the coronary heart.Model 1: Covariates unadjusted. Model 2: Adjust for age, gender, race, high blood pressure, high cholesterol, diabetes, stroke, smoking, drinking, and BMI. Model 3: Adjust for age (low, middle, high), gender, race, high blood pressure, high cholesterol, diabetes, stroke, smoking, drinking, and BMI (low, middle, high)

	Non-adjusted	Adjust I	Adjust II
**THEOPHYLLINE**			
Control group(n=5005)	Ref.	Ref.	Ref.
Heart disease group (n=222)	-0.4 (-0.9, 0.1) 0.0923	-0.8 (-1.5, -0.1) 0.0196	-0.7 (-1.5, -0.0) 0.0412
**PARAXANTHINE**			
Control group(n=5005)	Ref.	Ref.	Ref.
Heart disease group (n=222)	-4.8 (-8.0, -1.7) 0.0028	-5.1 (-8.4, -1.8) 0.0023	-4.1 (-7.3, -0.8) 0.0135
**THEOBROMINE**			
Control group(n=5005)	Ref.	Ref.	Ref.
Heart disease group (n=222)	-2.5 (-8.2, 3.2) 0.3925	-0.1 (-5.9, 5.7) 0.9689	-0.1 (-5.9, 5.7) 0.9770
**CAFFEINE**			
Control group(n=5005)	Ref.	Ref.	Ref.
Heart disease group (n=222)	1.2 (-0.4, 2.8) 0.1327	-0.9 (-2.5, 0.8) 0.2966	-0.5 (-2.1, 1.1) 0.5534

### Subgroup analysis

Subgroup variables: including sex, age, race, high blood pressure, high cholesterol level, diabetes, BMI Tertile, stroke, smoking, alcohol drinks in 1 Yr, MET score (MET score<4: it indicates severe functional restriction, which is related to significantly increased risk of cardiovascular disease. MET score 4-6: it indicates moderate functional restriction, which may be significant dyspnea or fatigue when completing moderate-intensity activities (such as short-distance walking and light labor). MET score>8 usually indicates a lower risk of cardiovascular disease. Sample size (N): The number of samples in each subgroup. Concentration difference and statistical significance: In each subgroup, display the difference (95% confidence interval and p-value) between the heart disease group and the control group in the concentration of four compounds.

Through stratified analysis,as shown Table 5 , we found that in most subgroups, the concentration difference of theophylline did not reach statistical significance (p > 0.05). For example, in the population without hypertension, the difference was -1.3 (95% CI: -4.8, 2.2), but still not significant (p = 0.4758). There was no significant difference in the concentration of theophylline among the groups. The overall difference in Paraxanthine was significant and consistent across multiple subgroups: gender: female (difference -7.4, p = 0.0379), male (difference -4.8, p = 0.0538). Race: Non-Hispanic White (difference -7.3, p = 0.0034); There was no significant difference in other groups. Hypertensive patients: The difference is -9.7, p = 0.0299. In the high BMI group, the difference is -6.5, p = 0.0457. No smoking history: difference of -7.0, p = 0.0265, MET < 4 points: the difference is -6.7, p = 0.0362.

**Table 5:** – Subgroup analysis. This table presents the stratified analysis results of theophylline, paraxanthine, theobromine, and caffeine in different subgroups, including effect values, 95% confidence intervals, and p-values. Through stratified analysis, differences in compound concentrations and their significance can be observed among different characteristic groups

Sub-group	N	THEOPHYLLINE	PARAXANTHINE	THEOBROMINE	CAFFEINE
**SEX**					
Male	2543	-0.4 (-2.7. 1.8) 0.7161	-4.8 (-9.8. 0.1) 0.0538	0.4 (-6.0. 6.8) 0.9105	2.2 (0.6. 3.8) 0.0081
Female	2684	-0.7 (-1.7. 0.4) 0.2290	-7.4 (-14.5. -0.4) 0.0379	-6.7 (-16.5. 3.1) 0.1791	-0.5 (-3.1. 2.1) 0.7220
**AGE YEAR Tertile**					
Low (18≤age≤39)	1685	-0.9 (-3.6. 1.9) 0.5380	-11.0 (-37.5. 15.4) 0.4137	-17.7 (-53.9. 18.5) 0.3381	-3.9 (-11.3. 3.6) 0.3106
Middle (39<age≤57)	1740	-0.6 (-1.7. 0.5) 0.2582	-2.0 (-13.3. 9.3) 0.7259	-8.8 (-23.3. 5.8) 0.2373	0.9 (-2.6. 4.4) 0.6177
High (58≤age≤80)	1802	-0.8 (-3.3. 1.7) 0.5434	-3.6 (-7.7. 0.5) 0.0882	2.0 (-3.8. 7.8) 0.5072	0.3 (-1.5. 2.1) 0.7589
**RACE**					
Mexican American	717	1.7 (-0.0. 3.4) 0.0531	3.1 (-12.4. 18.5) 0.6971	3.7 (-12.0. 19.4) 0.6447	9.0 (3.9. 14.1) 0.0006
Other Hispanic	522	0.0 (-1.4. 1.5) 0.9699	1.2 (-12.3. 14.8) 0.8565	-5.4 (-22.1. 11.3) 0.5295	3.3 (-1.0. 7.6) 0.1348
Non-Hispanic White	2257	-1.1 (-3.6. 1.4) 0.3858	-7.3 (-12.2. -2.4) 0.0034	-4.9 (-12.3. 2.5) 0.1936	-0.9 (-2.8. 1.1) 0.3745
Non-Hispanic Black	1100	-0.7 (-1.6. 0.2) 0.1460	-7.7 (-18.3. 2.9) 0.1538	-7.7 (-22.1. 6.7) 0.2933	-0.0 (-3.1. 3.0) 0.9936
Other Race - Including Multi-Racial	631	-0.3 (-1.9. 1.2) 0.6714	-2.6 (-19.8. 14.6) 0.7695	-7.0 (-27.6. 13.6) 0.5071	1.7 (-2.8. 6.1) 0.4658
**HIGH BLOOD PRESSURE**					
Yes	1908	0.1 (-0.5. 0.7) 0.8032	-1.4 (-5.6. 2.9) 0.5379	1.5 (-4.2. 7.2) 0.6026	1.5 (-0.2. 3.3) 0.0869
No	3310	-1.3 (-4.8. 2.2) 0.4758	-9.7 (-18.5. -1.0) 0.0299	-7.2 (-19.2. 4.7) 0.2365	-1.5 (-4.3. 1.3) 0.2977
Do not know	9				
**HIGH CHOLESTEROL LEVEL**					
Yes	1715	-0.2 (-0.7. 0.3) 0.4859	-3.7 (-8.3. 1.0) 0.1196	-1.6 (-8.3. 5.0) 0.6333	1.0 (-0.8. 2.8) 0.2794
No	2943	-0.7 (-3.8. 2.4) 0.6428	-7.0 (-15.1. 1.1) 0.0924	-3.6 (-14.0. 6.7) 0.4927	-0.4 (-3.1. 2.4) 0.7814
Do not know	34	-0.5 (-4.8. 3.9) 0.8378	-13.2 (-57.1. 30.7) 0.5605	-14.6 (-68.1. 38.9) 0.5969	0.0 (-12.4. 12.4) 0.9987
Ausente	535				
**DIABETES**					
Yes	615	-0.4 (-1.2. 0.4) 0.3501	-6.8 (-13.6. 0.0) 0.0513	0.1 (-8.5. 8.7) 0.9758	-1.0 (-3.6. 1.7) 0.4773
No	4480	-0.2 (-1.8. 1.4) 0.7934	-2.6 (-7.6. 2.3) 0.2998	-0.9 (-7.5. 5.7) 0.7801	2.1 (0.4. 3.8) 0.0165
Borderline	127	-3.2 (-22.6. 16.3) 0.7500	-8.0 (-28.2. 12.3) 0.4417	-11.2 (-50.9. 28.6) 0.5830	-0.6 (-9.3. 8.0) 0.8841
Do not know	5				
**BMI Tertile**					
Low (13.4≤IMC≤25.69)	1724	-1.0 (-5.1. 3.0) 0.6270	-5.9 (-14.0. 2.2) 0.1543	-2.6 (-13.4. 8.2) 0.6394	-0.6 (-3.2. 2.1) 0.6778
Middle (25.7≤IMC≤30.59)	1750	-0.2 (-2.1. 1.7) 0.8438	-2.9 (-9.6. 3.8) 0.3952	-0.5 (-9.6. 8.5) 0.9080	1.9 (-0.6. 4.3) 0.1384
High (30.7≤IMC≤82.1)	1753	-0.3 (-1.0. 0.4) 0.4031	-6.5 (-12.8. -0.1) 0.0457	-4.9 (-13.6. 3.7) 0.2616	1.5 (-0.7. 3.7) 0.1743
**STROKE**					
Yes	167	-1.0 (-3.8. 1.7) 0.4676	-4.6 (-13.8. 4.6) 0.3249	-6.3 (-17.6. 5.0) 0.2746	-1.1 (-5.5. 3.3) 0.6228
No	5056	-0.3 (-1.8. 1.1) 0.6445	-4.1 (-8.4. 0.3) 0.0663	-1.2 (-7.0. 4.6) 0.6889	1.3 (-0.2. 2.8) 0.0862
Do not know	4				
**SMOKED AT LEAST 100 CIGARETTES IN LIFE**					
Yes	2296	-0.1 (-1.6. 1.4) 0.8906	-4.8 (-10.1. 0.6) 0.0805	1.0 (-5.4. 7.5) 0.7546	1.8 (-0.1. 3.7) 0.0675
No	2930	-1.1 (-3.5. 1.3) 0.3891	-7.0 (-13.1. -0.8) 0.0265	-6.0 (-15.1. 3.1) 0.1957	-0.3 (-2.4. 1.8) 0.7948
Do not know	1				
**AT LEAST 12 ALCOHOL DRINKS IN 1 YR**					
Yes	3434	-0.4 (-2.2. 1.3) 0.6268	-4.7 (-9.7. 0.3) 0.0677	-1.4 (-7.9. 5.0) 0.6600	0.9 (-0.9. 2.7) 0.3292
No	1303	-0.5 (-3.2. 2.1) 0.6935	-5.5 (-12.8. 1.8) 0.1398	-4.9 (-15.9. 6.1) 0.3797	1.8 (-0.4. 4.0) 0.1138
Do not know	2				
Missing	488	-0.5 (-2.0. 1.0) 0.5035	-7.1 (-22.3. 8.1) 0.3581	-5.8 (-29.3. 17.7) 0.6281	1.7 (-3.3. 6.6) 0.5120
**MET**					
<4	1355	-0.6 (-2.8. 1.7) 0.6099	-6.7 (-12.9. -0.4) 0.0362	-4.8 (-12.8. 3.2) 0.2381	1.6 (-0.8. 4.0) 0.1847
4-8	1462	-0.5 (-1.5. 0.4) 0.2511	-3.6 (-10.3. 3.0) 0.2818	2.0 (-6.4. 10.5) 0.6361	-0.1 (-2.4. 2.2) 0.9365
≥8	2410	-0.0 (-3.1. 3.1) 0.9958	-1.3 (-9.3. 6.6) 0.7388	-0.7 (-11.9. 10.5) 0.8991	1.8 (-0.8. 4.4) 0.1730

Therefore, the concentration of xanthine was significantly lower in most groups than in the control group, and this difference was more prominent in subgroups of females, race, and some health characteristics (such as hypertension and smoking, BMI index, and MET score). The concentration difference of theobromine did not reach statistical significance in all subgroups (p > 0.05). The difference in theobromine concentration between the heart disease group and the control group was not statistically significant and may have a weak relationship with heart disease. Caffeine showed differences in some subgroups, in gender, the difference for men was 2.2 (p = 0.0081), while there was no significant difference for women (p = 0.7220), in Mexican-American race there was a difference of 9, p = 0.0006, there was no significant difference in other groups. For patients without diabetes, the difference was 2.1, p = 0.0165. Therefore, the difference in caffeine concentration between men and Mexican-American and non-diabetic patients may be more significant, but there is no consistency in the overall group and the other groups.

## Discussion

Based on the analysis of this 6-year large-scale cross-sectional study, we created a Central Illustration that clearly summarizes the main data of the article. The figure shows that there are significant baseline characteristic differences between the control group and the CHD group, particularly that individuals in the CHD group are generally older and have higher prevalence rates of hypertension, hypercholesterolemia, diabetes, stroke, smoking, and alcohol use. The analysis of urinary caffeine levels and CHD incidence revealed a positive correlation, especially in the Mexican American population, where this association was more pronounced (odds ratio [OR] = 1.03, 95% confidence interval [CI]: 1.03–1.05, p = 0.0081). Specifically, for every 1 μmol/L increase in urinary caffeine levels, the risk of CHD increased by 1.03 times, with statistical significance (p < 0.01). However, no significant association was found between urinary caffeine metabolites (such as theobromine and theophylline) and CHD. The theophylline and hypoxanthine concentrations were significantly lower in the CHD group compared to the control group. Subgroup analysis further showed significant differences in hypoxanthine levels across different sex, age, race, hypertension, and smoking subgroups, particularly in women, those with hypertension, and smokers. No significant differences in the concentrations of theobromine and caffeine were observed across most subgroups. These results suggest that the relationship between urinary caffeine and CHD may be influenced by factors such as race and other physiological factors and that the caffeine metabolite hypoxanthine may be associated with cardiovascular health. This study provides valuable reference for clinical and basic research, aiding in the identification and intervention of risk factors for CHD.

Previous studies have shown that coffee is positively correlated with the risk of CHD (OR 1.35, 95% CI 1.05-1.73; P=0.017) and the occurrence of hypertension.^9 , 15^Moderate or excessive consumption of coffee is associated with an increased inflammatory process, which plays a crucial role in the development of CHD.^16^Excessive consumption of coffee, tea, and caffeine may increase the risk of all-cause mortality and cardiovascular disease death in patients with cardiovascular disease.^17^Unfiltered coffee appears to increase levels of serum triglycerides, cholesterol, and low-density lipoprotein (LDL).^18 , 19^

However, some studies have also shown that drinking coffee is safe and does not increase the risk of death. The impact of different coffee preparations on cardiovascular outcomes and survival rates varies. Decaffeinated coffee, coffee powder, and instant coffee are associated with a significant reduction in the incidence and mortality of cardiovascular disease.^20^Grinding and instant coffee without decaffeination are associated with a lower risk of cardiac arrhythmias. This suggests that the diversity of sources and intake of caffeine may affect research results.^21^

In conclusion, our study used caffeine and its metabolites in urine as indicators of true coffee intake and found that the level of caffeine in urine was positively correlated with the incidence of CHD (OR=1.54, 95% CI: (1.04, 2.3), p = 0.0318), supporting the reliability of our research method. Additionally, we found that the level of paraxanthine was negatively correlated with the incidence of CHD. The risk of death for those who drank small or large amounts of coffee was reduced compared to those who did not drink coffee. Those who drank a small amount of coffee (1-2 cups/day) and a large amount of coffee (>2 cups/day) had a lower risk of death, with heavy coffee drinkers benefiting the most.

Several factors may explain these results. Firstly, coffee is a complex compound containing over 100 bioactive ingredients, with caffeine being the most well-known.^22 , 23^Acute intake of caffeine can activate the sympathetic nervous system by inhibiting phosphodiesterase, increasing cytoplasmic calcium, and releasing norepinephrine/adrenaline, which may increase cardiovascular reserve and maintain coronary artery blood flow, thereby reducing the occurrence of CHD. Secondly, the antagonistic effect of caffeine on adenosine may weaken the expected increase in myocardial blood flow during exercise.^24^Thirdly, caffeine intake has been found to regulate risk factors for CHD, especially in non-smokers. Drinking coffee may have a potentially beneficial effect on preventing coronary artery calcification.^25^Fourthly, the antioxidant effect of caffeine can reduce endothelial cell damage and lower the risk of CHD. Fifth, phenolic compounds in coffee, especially hydroxycinnamic acid, have a protective effect on the cardiovascular system, mainly achieved through anti-inflammatory properties and antioxidant activity. In contrast, short-term consumption of unfiltered coffee may increase serum triglycerides, cholesterol, and LDL levels.^18^

In this large-scale cross-sectional study, we observed a negative correlation between urinary caffeine levels and the incidence of CHD in Mexican Americans, but no similar association was found in other ethnic groups. This difference may be related to the varying incidence rates of CHD among different racial groups.^26^Additionally, genetic variation may also influence individual responses to caffeine. For example, the study by Happonen et al.^27^indicated that certain gene polymorphisms (such as the COMT gene) may modulate the effects of caffeine on cardiovascular events. The literature^28^points out that elevated plasma caffeine concentrations in men are associated with reduced stress myocardial blood flow and myocardial blood flow reserve, while similar hemodynamic responses were not observed in women. However, in our study, no sex-related differences were noted. Therefore, more high-quality, large-sample, randomized controlled studies are needed to include genetic factors and further elucidate the modulatory effects of these genetic polymorphisms on the relationship between caffeine and cardiovascular health.

In regression analysis, we conclude that the concentrations of theophylline and hypoxanthine in the heart disease group were significantly lower than those in the control group, especially after adjustment, which still showed statistical significance, suggesting that these compounds may be associated with heart disease. There was no significant difference in the concentrations of theobromine and caffeine between the two groups, which may not be directly related to heart disease. Theophylline is a secondary product of caffeine metabolism. The decrease in its concentration may reflect impaired metabolic function; for example, heart disease patients may experience a decrease in liver function or overall metabolic capacity, leading to a reduction in theophylline production. In addition, it may also be affected by inflammation or oxidative stress: cardiovascular disease is often accompanied by systemic inflammation or oxidative stress, which may interfere with the metabolic pathway of theophylline. The adjusted results indicate a significant increase in theophylline concentration, suggesting that other confounding factors, such as age, gender, BMI, etc., may mask the true association between theophylline and heart disease in the unadjusted model. The significant decrease in theophylline concentration suggests its potential diagnostic value. In the future, genomic data (such as the CYP1A2 gene) can be combined to verify further whether changes in theophylline concentration can predict the risk of heart disease or serve as efficacy monitoring indicators in cardiovascular therapy. In all models, xanthine levels were significantly lower in the heart disease group compared to the control group (difference range: -4.1 to -5.1, p<0.05). The significant difference in xanthine levels before and after adjustment is consistent, indicating a more direct association with heart disease and less susceptibility to confounding factors and suggesting its potential as an independent metabolic marker for heart disease. There was no significant difference in the concentrations of theobromine and caffeine between the heart disease group and the control group, which may not directly participate in cardiovascular, metabolic processes, or their differences may be masked by diet and lifestyle habits. In the future, paraxanthine can be included in the early screening biomarker library for heart disease, and personalized diagnosis can be carried out by combining genomic data. Develop targeted prevention and control strategies based on the regulatory effects of theophylline and hypoxanthine, taking into account gender, race, and metabolic status.

Subgroup analysis showed that paraxanthine was the only compound significantly reduced in most groups, especially in subgroups such as females, hypertensive patients, high BMI, and low MET score populations, suggesting that it may be a potential key biomarker for heart disease. Paraxanthine is the main product of caffeine metabolism and has certain anti-inflammatory and lipid metabolism-regulating effects. The significant decrease in paraxanthine concentration in patients with heart disease may reflect the characteristics of elevated inflammatory factors or blocked metabolic pathways in cardiovascular disease. In patients with hypertension and high BMI, there is a higher level of metabolic stress, and the reduction of paraxanthine may be related to lipid metabolism disorders, weakened liver function, and systemic inflammatory status. The significant reduction of paraxanthine in women, hypertensive patients, and patients with severe functional impairment suggests possible gender differences and the exacerbating effect of hypertension on the metabolic characteristics of heart disease. The significance of Non-Hispanic White race may reflect differences in metabolic enzyme activity, lifestyle habits, and disease susceptibility among different races. In future research, we plan to conduct large-scale cohort studies to further validate the effectiveness of xanthine as a biomarker for heart disease. There was no statistically significant difference in the concentration of theophylline and theobromine, indicating a weak association with heart disease. Theophylline is mainly an intermediate product of caffeine metabolism, and its metabolism is greatly influenced by genes and liver enzyme activity (such as the polymorphism of the CYP1A2 gene). Due to the fact that these metabolic processes do not directly participate in cardiovascular pathophysiological changes, their concentration changes may not have a significant response to heart disease. In addition, theophylline has a longer half-life in the human body, which may mask the impact of transient concentration fluctuations on heart disease. Cocoa alkaloids are a secondary metabolite of caffeine, with low metabolic efficiency and relatively stable concentrations in the human body, which may not significantly reflect metabolic changes related to heart disease. The concentration differences of caffeine are only significant in a few specific subgroups, and the overall trend is inconsistent, which may reflect a weak correlation with heart disease or interference from other factors. The significant increase in caffeine concentration in males may be related to lifestyle (such as coffee consumption habits) and higher metabolic rates in males, while estrogen levels in females may interfere with caffeine metabolism, thereby reducing the differences. The significantly elevated concentration in Mexican American may be associated with race-specific polymorphisms in caffeine metabolizing enzyme genes, as well as cultural and dietary influences. The concentration of caffeine in patients without diabetes increased significantly, suggesting that diabetes may affect the clearance rate of caffeine through metabolic disorders. Therefore, a significant decrease in xanthine may become a metabolic marker for heart disease, which can aid in early screening and intervention. The discovery of specific subgroups can help develop personalized strategies for preventing and controlling heart disease.

Our research has the following advantages. Firstly, the study used NHANES data for large sample analysis, effectively reducing bias caused by insufficient sample size. Secondly, we stratified the data by gender, age, and race and considered multivariable confounding factors such as hypertension and BMI for multivariable regression analysis. This method allows us to more accurately explore the relationship between caffeine and caffeine metabolites in urine and the incidence of CHD. Thirdly, we used the levels of caffeine and caffeine metabolites in urine as exposure variables to avoid the effects of recall bias, misclassification, and differences in food types rather than relying on caffeine intake reported in questionnaires. Fourthly, by constructing different models and analyzing multivariable covariates, we obtained more reliable results on the relationship between caffeine and its metabolites and CHD.

However, this study also has limitations. Firstly, as this is a cross-sectional study, it is not possible to determine the causal relationship between caffeine metabolites and CHD. Secondly, the study subjects mainly come from the United States and may not represent the global population. Thirdly, due to the stratification method of NHANES data and the low incidence rate of CHD, the sample size of the CHD group is small, which may lead to some bias in the results. Therefore, further longitudinal studies and multi-regional, multi-center research are needed to validate these findings and explore the exact relationship between caffeine and its metabolites and CHD.

## Conclusion

In this study, we found that urinary paraxanthine levels were inversely associated with the risk of CHD. Meanwhile, urinary caffeine levels were positively associated with CHD rates in Mexican Americans, but this association was not found in other groups. Overall, urinary caffeine levels were positively associated with CHD incidence, but there was no significant association in sex-stratified analyses. The results of this study can provide a reference for clinical prediction and diagnosis and are of great significance in clinical practice, but further large-scale prospective studies are needed to verify these conclusions.

## *Supplemental Materials

For additional information, please click here.
